# Chromogranin B acts as a neuronal paracrine factor to trigger oligodendrocyte apoptosis

**DOI:** 10.1371/journal.pone.0358362

**Published:** 2026-09-21

**Authors:** Nanako Yamada, Momona Yamada, Reina Ono, Binri Sasaki, Sakurako Abe, Tomoka Aoki, Akira Yoshimoto, Ryunosuke Ohkawa, Nobuharu Suzuki

**Affiliations:** 1 Department of Clinical Bioanalysis and Molecular Biology, Graduate School of Medical and Dental Sciences, Institute of Science Tokyo/Tokyo Medical and Dental University (TMDU), Tokyo, Japan; 2 Department of Molecular and Cellular Biology, Graduate School of Medical and Dental Sciences, Institute of Science Tokyo/TMDU, Tokyo, Japan; University of Texas Medical Branch at Galveston, UNITED STATES OF AMERICA

## Abstract

Various soluble factors, such as inflammatory cytokines and autoantibodies secreted by activated microglia, macrophages, and astrocytes, are known to promote death of oligodendrocyte (OL) lineage cells, including OLs and OL precursor cells (OPCs), in the central nervous system during demyelination of multiple sclerosis. However, the role of factors secreted by neurons in OL lineage cell death remains underexplored. Chromogranin B (Chgb), a critical protein for secretory granule formation in neuroendocrine cells, is also specifically expressed and secreted from neurons. In this study, we investigated the effect of extracellular Chgb on OL lineage cells. We found that treatment with recombinant mouse or human Chgb significantly reduced the population of both mature OLs and OPCs by inducing apoptosis. This Chgb-induced apoptosis was effectively suppressed by an anti-Chgb antibody. Furthermore, in neuron-OPC/OL cocultures, endogenous Chgb secreted by neurons similarly promoted OL apoptosis, an effect that was also neutralized by the anti-Chgb antibody. Collectively, these results identify Chgb as a neuronal paracrine factor that triggers apoptotic death in OL lineage cells. Our findings provide new insights into the regulation of OL lineage cell death by neuronal secretion factors.

## Introduction

In the central nervous system (CNS), myelin is essential for the physical and physiological support of axons. Axon demyelination results in several diseases, multiple sclerosis (MS) being one of the most prevalent. MS is characterized by reduced functions in mobility, balance, vision, and cognition as well as sensory abnormalities [1]. Currently, MS represents a significant global health burden, affecting over 2 million people worldwide [2]. Furthermore, recent epidemiological data indicate a rising incidence of the disease, with diagnosis occurring at increasingly younger ages [3]. Given this expanding clinical burden, identifying therapeutic strategies to protect axons from demyelination remains a critical and active area of research [4]. Central to this protective mechanism is oligodendrocyte (OL) lineage cells.

Neuronal axons are myelinated by OLs, which wrap them with multiple layers of thin sheet-like plasma membranes and provide substrates for their ATP production, such as pyruvate and lactate [5]. This enables fast and efficient conduction of the neural action potential and maintains axonal homeostasis [5]. Conversely, neurons express and secrete various molecules to promote proliferation, survival and differentiation of OL precursor cells (OPCs) and OLs (e.g., insulin-like growth factor I, platelet-derived growth factor factor-A, semaphorins, and neurotransmitters) [6]. Whereas some neuronal membrane proteins possess inhibitory effects on OL lineage cell differentiation and myelination (e.g., LINGO-1, Delta1/Jagged-1, PSA-NCAM), their signaling is necessary for the progression of these stages with proper timing [6]. Therefore, OL lineage cells and neurons have a mutually reciprocal relationship.

In demyelinating lesions, inflammatory cytokines, such as interleukins, interferons, and autoantibodies against myelin proteins, have been identified as promoters of OL death and demyelination [7]. These extracellular factors are primarily secreted from immune cells, including activated microglia/macrophages, and activated astrocytes [7]. However, while the impact of immune-derived factors has been extensively studied, the role of soluble factors secreted by neurons in promoting demyelination remains largely unknown. Recent reports on MS and experimental animal models of autoimmune encephalomyelitis (EAE) studies indicate a contradiction: myelinated axons exhibit greater degeneration than unmyelinated ones. This occurs because myelin sheaths of dysfunctional OLs lose their ability to metabolically support the axons while acting as a physical barrier, preventing the uptake of metabolites from the extracellular environment [8]. Thus, to survive this metabolic starvation, neurons may utilize an intrinsic clearance mechanism to remove dysfunctional myelin by releasing factors that induce OL death.

The granin family, comprising chromogranins and secretogranins, represents a unique class of acidic secretory proteins. These proteins are stored in dense core vesicles, which are the reservoirs for neurotransmitters, neuropeptides, and hormones, and their functions in neurons and neuroendocrine cells are well-established [9]. Chromogranin B (Chgb), a major member of this family, is a hydrophilic glycoprotein characterized by a high proportion of acidic amino acids residues (~30%) and post-translational modifications, including sulfation and phosphorylation. In the context of demyelinating diseases, Chgb expression appears complex. While Chgb levels were significantly reduced in the cerebrospinal fluid of MS patients [10], studies in EAE mice revealed a distinct temporal pattern [11]. During the late symptomatic stage of EAE, Chgb secretion was impaired, leading to intracellular accumulation and a decline in extracellular levels. However, before symptom onset, Chgb secretion is upregulated, resulting in elevated extracellular levels [11]. Despite these observations, the specific role of the extracellular Chgb, particularly its paracrine effect on myelinating OLs and surrounding OPCs during the early phase of demyelination, remains unknown.

In this study, we therefore aimed to elucidate the effect of extracellularly secreted Chgb on OL lineage cells. We found that recombinant mouse Chgb (mChgb) acts as a soluble factor that significantly decreases the number of OPCs and OLs in primary cultures by inducing apoptosis. We further observed that this Chgb-mediated apoptosis was significantly inhibited by an anti-Chgb antibody. Given the sequence divergence between mouse and human Chgbs, we additionally examined the activity of recombinant protein of human Chgb (hChgb). It exerted a cytotoxic effect on OPCs/OLs survival comparable to that of mChgb. Furthermore, we demonstrated that endogenous Chgb released from primary neurons also promoted apoptotic OL lineage cell death in neuron-OPC/OL cocultures. Taken together, our results indicate that Chgb functions as a neuronal paracrine factor that induces apoptosis in OL lineage cells.

## Materials and methods

### Animals

Wister rats (Clea Japan) at the age of embryonic day (E)15 and postnatal day (P)1 or P2 were used for primary cultures. These rat embryos and pups were euthanized by decapitation, which followed the guideline from the American Veterinary Medical Association (AVMA). The humane endpoints were the completion of their life at the stages (day 15 of pregnancy/embryo, P1, and P2) by the appropriate methods. None of these rats died or displayed specific signs of severe suffering or distress before the moment of euthanasia. The duration of the experiments for euthanasia was 1 second at the decapitation of one pup or embryo. Totally, 60 of E15 embryos and 90 of P1 or P2 pups were euthanized and used for the experiments in this study. We monitored the health conditions of the rats every day. We also considered the animal welfare throughout this study by following the guidelines, although none of rats exhibited specific signs of suffering or distress. We took the special training in animal care and handling by veterinarians in the Animal Research Facility of the Institute of Science Tokyo/Tokyo Medical and Dental University (TMDU). All procedures for genetic recombination experiments and animal experiments were approved by the internal committees of the Institute of Science Tokyo/TMDU (Approval No: G2024-004C3 and A2025-042C), followed the Animal Care Standards of the Institute of Science Tokyo/TMDU in the management and handling of experimental animals, and complied with the ARRIVE guidelines. An equal number of male and female animals was used for the experiments.

### Plasmid construction and preparation of recombinant proteins

For the preparation of mChgb, cDNA from the spinal cord of P3 C57BL/6 mouse was used as a template for PCR to amplify the coding sequence, including the endogenous signal peptide, of *Chgb* with the primers (forward: 5’-GACTAGTCACCATGCAGCCGGCTATGCTCCTC-3’; reverse: 5’-GATAAGAATGCGGCCGCCGCCCCGCTGGCTGAACTTTTC-3’). The amplified DNA fragment was inserted into pEF1/V5-His B (ThermoFisher Scientific) by digestion with *Spe* I (Takara Bio) and *Not* I (Takara Bio) and a ligation with T4 DNA ligase (New England Biolabs). For the preparation of hChgb, cDNA from 293T cells and primers (forward: 5’-GAGCTCGGATCCACTAGTCACCATGCAGCCAACGCTGCTTCTC-3’; reverse: 5’- CTAGACTCGAGCGGCCGCAGCCCCTTTGGCTGAATTTCTC -3’) were used for PCR to amplify the coding sequence of *CHGB*, containing its signal peptide. In addition, PCR was performed using the following primers (forward: 5’-GCGGCCGCTCGAGTCTAGAG-3’; reverse: 5’-ACTAGTGGATCCGAGCTCGGTACCA-3’) and pEF1/V5-His B as a template. The two fragments obtained by the PCRs were assembled using the NEBuilder HiFi DNA Assembly Cloning Kit (New England Biolabs). For the expression and purification of both mChgb and hChgb, the previous protocol was followed [12]. The concentration of the protein samples was measured with the Qubit Protein Assay Kit (ThermoFisher Scientific).

### Western blotting

Western blotting was performed as previously described [12]. Protein samples were dissolved in sodium dodecyl sulfate-polyacrylamide gel electrophoresis sample buffer (FUJIFILM Wako Pure Chemical) containing 20 mM dithiothreitol (ThermoFisher Scientific) and were denatured for 10 minutes at 95°C. The denatured samples were analyzed using an 8% polyacrylamide gel. After electrophoresis, the proteins were transferred to a polyvinylidene fluoride membrane (Cytiva). The membrane was blocked with 5% skim milk in tris-buffered saline. Mouse Anti-V5 Antibody (ThermoFisher Scientific) and rabbit anti-chromogranin B antibody (Proteintech) were used as primary antibodies, and anti-mouse IgG-HRP (Cell Signaling Technology) and anti-rabbit IgG-HRP (Cell Signaling Technology) were used as secondary antibodies, respectively. For the detection of protein bands, ImmunoStar LD (FUJIFILM Wako Pure Chemical) was used.

### Preparation of primary OPCs and OLs

Cerebral cortices from P1 or P2 rats were dissected out and minced with a scalpel. Tissues were enzymatically digested using papain (Worthington Biochemical) and DNase I (Merck) for 20 minutes at 37°C. After centrifugation for 5 minutes at 800 rpm, the supernatant was removed. The cell pellet was suspended with DMEM20S, and the cell suspension was passed through a cell strainer (pore size: 70 μm; SPL life sciences). Cells were seeded at approximately 1.0 × 10^7^ cells per 75 cm^2^ flask (ThermoFisher Scientific) precoated with poly-D-lysine (PDL; Merck), and incubated at 37°C for 3 or 4 days. After that, the medium was changed, and cells were cultured at 37°C for additional 2 days. At the end of the culture, the culture flasks were shaken at 160 rpm on an orbital shaker for an hour at 37°C and the medium containing microglia was removed. The flasks were shaken again at 240 rpm for 22 hours at 37°C with fresh DMEM20S. The cell culture medium was collected and passed through a cell strainer with a mesh size of 70 μm. After mixing by inversion, 10 mL each was seeded on a Petri dish (TGK) and incubated at 37°C for 15 minutes to let astrocytes attach to the dish. The medium containing OPCs was collected and centrifuged at 800 rpm for 10 minutes, and the supernatant was removed and centrifuged again under the same conditions. The precipitated cells from both centrifugations were collected and suspended in OPC medium. The isolated cells were plated on a 12-well slide (Matsunami Glass, 3 × 10^3^ cells/well) precoated with PDL (Day *in vitro* 0: DIV0). The medium of the OPC culture was changed to OPC medium to remove fetal bovine serum (FBS) one day after OPCs were plated (DIV1). For the analysis of OPCs, we continued to culture the cells for 3 days in the same medium (DIV4 for OPC). For the analysis of OLs, OPC medium was changed to OL medium on DIV2, and then, their differentiation was induced to OLs for 3 days (DIV5 for OL). We confirmed the expression of NG2 and GalC, markers for OPCs and OLs, respectively, at DIV2, DIV4 for OPC, and DIV5 for OL in the absence of Chgb (S1 Fig).

DMEM20S: 4 mM L-glutamine (Merck), 1 mM pyruvate (Merck), 20% FBS (MP Biomedicals), and 100 U/mL penicillin and 100 μg/mL streptomycin (ThermoFisher Scientific) in Dulbecco’s Modified Eagle Medium (DMEM; high glucose, no glutamine; ThermoFisher Scientific).

Basal chemically defined medium (BDM): 4 mM L-glutamine, 1 mM pyruvate, 0.1% bovine serum albumin (BSA; Merck), 50 μg/mL apo-transferrin (Merck), 5 μg/mL insulin (Merck), 30 nM sodium selenite (Merck), 10 nM D-biotin (Merck), 10 nM hydrocortisone (Merck), and 100 U/mL penicillin and 100 μg/mL streptomycin in DMEM.

OPC medium: BDM containing 10 ng/mL platelet-derived growth factor-AA (ThermoFisher Scientific) and 10 ng/mL basic fibroblast growth factor (ThermoFisher Scientific).

OL medium: BDM containing 40 ng/mL triiodo-thyronine (Merck), 10 ng/mL ciliary neurotrophin factor (ThermoFisher Scientific), and 50 ng/mL N-acetyl-L-cysteine (Merck).

### Preparation of primary neurons

Dorsal root ganglions (DRGs) were collected from E15 rats and plated on a 12-well slide (2 or 3 DRGs/well) precoated with PDL (DIV0). On the next day, half of the culture medium (Neuron medium) was replaced with fresh medium containing AraC (Merck) (Final concentration of AraC: 10 µM) (DIV1). At day 6 (DIV6 for neuron), TNF-α (ThermoFisher Scientific) was added to the DRG culture at a replacement of half of the medium (Final concentration of TNF-α: 0.02 μg/mL). After 4 days (DIV10 for neuron), the samples of cell lysates and conditioned media were collected for Western blotting as described in the following section.

Neuron medium: Neurobasal medium (ThermoFisher Scientific) with 2% B-27 Supplement (ThermoFisher Scientific), 0.25 mM GlutaMAX Supplement (ThermoFisher Scientific), 0.25 mM L-glutamine solution, and 100 U/mL penicillin and 100 µg/mL streptomycin.

### Conditioned medium and cell lysate collection

DRG neurons were cultured in Neuron medium as described above and incubated at 37℃ for 10 days. At the end of the culture (DIV10 for neuron), the conditioned medium was collected, and DRG neurons were lysed with lysis buffer [1% NP-40 (FUJIFILM Wako Pure Chemical), PhosSTOP (Roche), EDTA-free Complete Protease inhibitor mixture (Roche), in tris-buffered saline] on ice. After centrifugation at 14,500 rpm for 10 minutes at 4°C, the supernatants were collected as cell lysates. The conditioned medium and cell lysate were used for Western blotting analyses.

### Preparation of coculture

Primary OPCs prepared from neonatal rats as described above were seeded on DRG neurons at day 6 of culture (DIV6 for neuron-OPC/OL). On day 7 (DIV7 for neuron-OPC/OL), the medium was changed to a mixture of Neuron medium and OPC medium at equal volumes. On day 8 (DIV8 for neuron-OPC/OL), the medium was changed to a mixture of Neuron medium and OL medium at equal volumes to induce differentiation from OPCs to OLs for 3 days (DIV11 for neuron-OPC/OL).

### Immunocytochemistry

OPCs were cultured in OPC medium for 3 days (DIV1-4 for OPC), and OLs were cultured in OL medium for 3 days (DIV2-5 for OL). mChgb or hChgb was added to the OPC/OL medium at 5 or 20 μg/mL at the start of culture in OPC/OL medium (DIV1 for OPC/DIV2 for OL). Coculture of neurons and OPCs/OLs was performed in a mixture of Neuron medium and OL medium at equal volumes for 3 days (DIV8-11 for neuron-OPC/OL) with or without 0.5 μg/mL of TNF-α. Cells were fixed in 4% paraformaldehyde (PFA; FUJIFILM Wako Pure Chemical) in PBS at room temperature (RT) for 10 minutes. They were washed with PBS and blocked at RT for 1 hour using Power Block (BioGenex), and incubated with a primary antibody in 1% BSA/PBS overnight at 4℃. After washing with PBS, they were labeled with a secondary antibody in 1% BSA for 50 minutes at RT, and washed again with PBS. The following primary antibodies were used: mouse anti-βIV-tubulin (Abcam), rabbit anti-NG2 antibody (Merck), mouse anti-NG2 antibody (Merck), rat anti-PDGFRα antibody (BD biosciences), mouse anti-GalC antibody (Merck), mouse anti-cleaved caspase-3 antibody (proteintech), rabbit anti-Tuj1 (Abcam), rabbit anti-myelin proteolipid protein (PLP; Merck), and mouse anti-CNP antibody (Merck). Secondary antibodies were used as follows: goat anti-mouse IgG-Alexa Fluor 488 (ThermoFisher Scientific), goat anti-mouse IgG-Alexa Fluor 594 (ThermoFisher Scientific), goat anti-rabbit IgG-Alexa Fluor 488 (ThermoFisher Scientific), goat anti-rabbit IgG-Alexa Fluor 594 (ThermoFisher Scientific), and goat anti-rat IgG-Alexa Fluor 594 (ThermoFisher Scientific). The samples were mounted with ibidi Mounting Medium (ibidi) and DAPI (Vector Laboratories) was used for nuclear staining. We often used mouse anti-βIV-tubulin antibody as a marker for both OPCs and OLs, since cell permeability was required for TUNEL assay and marker antibodies raised in rabbit were unusable in the inhibition experiments using the rabbit anti-Chgb antibody. After we tested three antibodies for OPCs in these conditions, the mouse anti-βIV-tubulin antibody was the most suitable among them. The mouse anti-βIV-tubulin antibody also detected OLs in the condition. (S2 Fig).

### TUNEL assay

Cell samples were prepared as described above. For the anti-Chgb antibody neutralizing experiments, the recombinant proteins (mChgb and hChgb) and the rabbit anti-chromogranin B polyclonal antibody (Proteintech) were added at 10 μg/mL (DIV1 for OPC/DIV2 for OL). Cells were fixed in 4% PFA in PBS at RT for 10 minutes and TUNEL assay was performed according to the instructions for the *In Situ* Apoptosis Detection Kit (Takara Bio). Immunostaining was performed as described above. TUNEL-positive rates out of total cells labeled with DAPI were measured. The primary antibodies used were mouse anti-Olig2 (Merck), mouse anti-βIV-tubulin, and rabbit anti-myelin PLP. For the functional blocking experiments with the rabbit anti-Chgb antibody, mouse anti-βIV-tubulin antibody, but not the rabbit anti-PLP antibody, was used to avoid cross-detection of anti-Chgb antibody. Secondary antibodies were goat anti-mouse IgG-Alexa Fluor 594 and goat anti-rabbit IgG-Alexa Fluor 594. The fluorescently labeled samples were mounted with ibidi Mounting Medium containing DAPI.

### Statistical analyses

All the experiments were independently performed at least 3 times. In some of the experiments, all the prepared cells, including cells for controls, were already more differentiated when plated, or were less differentiated after induction of differentiation. These were probably due to the different timing of their differentiation between the independent experiments, while they were cultured in the mixed glial culture from different rat cerebral cortexes. To correct for the variability in the differentiation conditions between independent experiments, cell numbers were normalized to the control group (set as 1.0). In addition, the number of independent experiments was added when the variability was high. Student’s *t*-test was used for the analyses in the experiments with two groups. One-way ANOVA followed by Dunnett’s post hoc test was used for the statistical analysis for multiple comparisons. Microsoft Excel and the R programming software were used for the statistical analyses. The statistical significance was defined as *: *p* < 0.05, **: *p* < 0.01.

## Results

### Extracellular mChgb reduces the population of OL lineage cells

**Fig 1:**
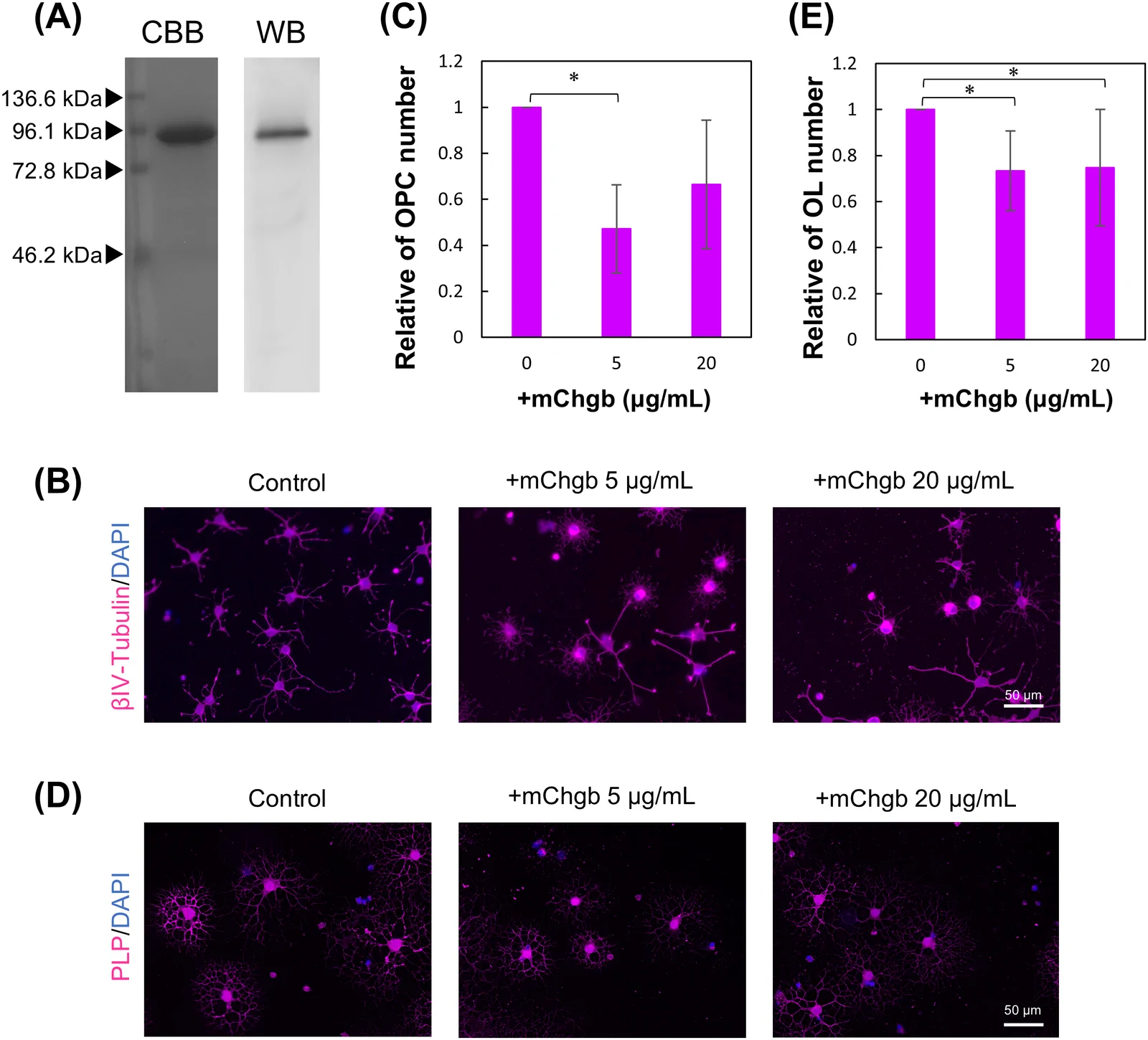
Decreased number of OPCs/OLs in the presence of mChgb. (A) Result of Coomassie brilliant blue staining (CBB) and Western blotting (WB) to detect mChgb. Representative immunostaining images of (B) βIV-tubulin (magenta) and (D) PLP (magenta) with DAPI staining (blue) in the primary (B) OPCs and (D) OLs, respectively, in the presence of 5 and 20 μg/mL of mChgb. The control cultures did not contain mChgb. Quantifications of the cell count in the primary (C) OPCs and (E) OLs under the condition with 5 and 20 μg/mL of mChgb. The numbers of independent experiments (biological replicates) are 5 in C and 9 in E. The statistical analyses were carried out as described in Materials and methods (*: *p* < 0.05). Error bars represent mean ± s.d.

We first investigated the effect of extracellular Chgb on the survival of OL lineage cells. To this end, we prepared mChgb and confirmed its high purity prior to use (Fig 1A; S3A, S3B Fig). To examine the effect of on OPCs, we treated primary OPCs cultures with mChgb at concentrations of 5 μg/mL and 20 μg/mL, and performed immunocytochemistry for βIV-tubulin, a specific tubulin isoform expressed throughout the OL lineage (Fig 1B). We confirmed that it was co-expressed with NG2, a standard marker for OPCs (S2A Fig), and explained the reason we chose this antibody in Materials and Methods. Quantitative analyses revealed that the number of OPCs was significantly decreased in the presence of 5 μg/mL mChgb (Fig 1C). A similar declining trend was observed at 20 μg/mL, although this did not reach statistical significance. Next, we assessed whether mChgb also affects differentiated OLs. We induced the differentiation of OPCs into mature OLs and exposed them to mChgb. Consistent with the results observed in OPC cultures, the number of mature OLs, identified by the myelin marker PLP, was significantly reduced in the presence of both 5 μg/mL and 20 μg/mL mChgb (Fig 1D, 1E).

### mChgb-induced cell loss is mediated by apoptosis

**Fig 2:**
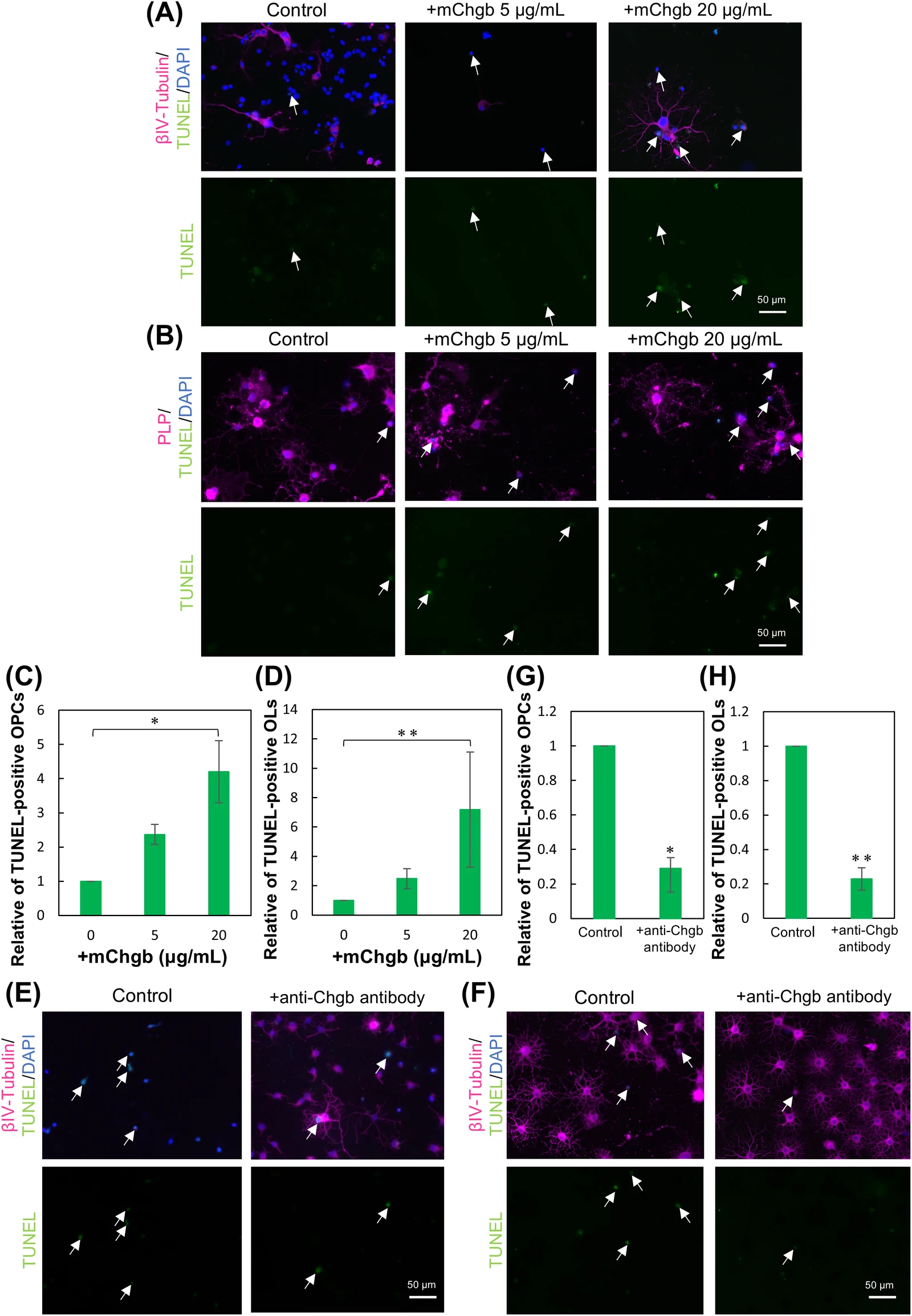
Induction of apoptosis of OPCs and OLs with mChgb. Representative images in TUNEL assay (green) combined with immunostaining of (A) βIV-tubulin (magenta) and (B) PLP (magenta) with DAPI staining (blue) in the primary (A) OPCs and (B) OLs, respectively, in the presence of 5 and 20 μg/mL of mChgb. The controls did not contain mChgb. Arrow: TUNEL-positive OPC or OL. Quantification of TUNEL-positive cell numbers in the primary (C) OPCs and (D) OLs under the condition with 5 and 20 μg/mL of mChgb. The numbers of independent experiments (biological replicates) are 5 in C and 3 in D. Representative images in TUNEL assay (green) combined with immunostaining of βIV-tubulin (magenta) and with DAPI staining (blue) in the primary (E) OPCs and (F) OLs in the presence of 10 μg/mL of mChgb and 10 μg/mL of the anti-Chgb antibody. The controls did not contain the anti-Chgb antibody. Arrow: TUNEL-positive OPC or OL. Quantification of TUNEL-positive cell numbers in the primary (G) OPCs and (H) OLs under conditions with 10 μg/mL of mChgb and 10 μg/mL of the anti-Chgb antibody. The numbers of independent experiments (biological replicates) are 3 in G and 3 in H. The statistical analyses were carried out as described in Materials and methods (*: *p* < 0.05, **: p < 0.01). Error bars represent mean ± s.d.

Given that the decrease in cell numbers was observed even in differentiated OLs, which are non-proliferative cells, we hypothesized that mChgb reduced the survival of OL lineage cells rather than inhibiting proliferation. To test this, we performed TUNEL assays to detect apoptotic death of these cells (Fig 2A, 2B). Quantitative analysis showed that the ratio of TUNEL-positive cells to total cells was significantly increased in both OPCs and OLs cultures in the presence of 20 μg/mL mChgb (Fig 2C, 2D). We also observed an increase in cleaved caspase-3-positive OPCs and OLs upon treatment with 20 μg/mL of mChgb (S4 Fig). These results suggest that the cell loss induced by mChgb is mediated by apoptosis.

To validate the specificity of this effect, we evaluated whether an anti-Chgb antibody could rescue the cells from apoptosis. We performed the TUNEL assays combined with immunocytochemistry for the lineage marker βIV-tubulin (Fig 2E, 2F). Co-expression of βIV-tubulin and PLP was confirmed in the differentiated culture conditions (S2D Fig). We found that the percentages of TUNEL-positive in both OPCs and OLs cultures were substantially attenuated by the addition of the anti-Chgb antibody (Fig 2G, 2H). These results demonstrated that the pro-apoptotic activity is specific to mChgb and validated this antibody as a functional neutralizing antibody.

### The cytotoxic effect is conserved in human Chgb

**Fig 3:**
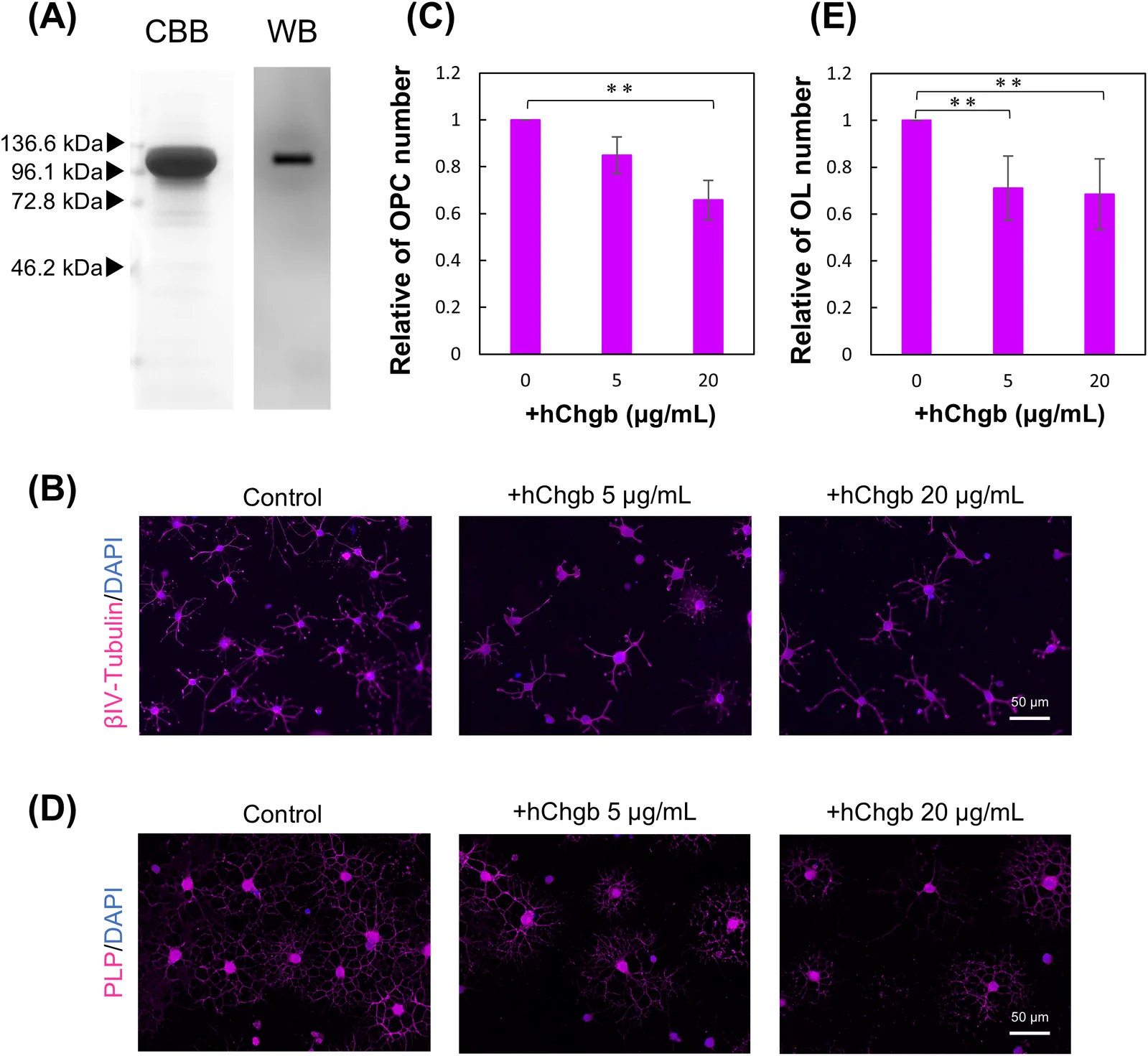
Decreased number of OPCs/OLs in the presence of hChgb. (A) Result of Coomassie brilliant blue staining (CBB) and Western blotting (WB) to detect hChgb. Representative immunostaining images of (B) βIV-tubulin (magenta) and (D) PLP (magenta) with DAPI staining (blue) in the primary (B) OPCs and (D) OLs, respectively, in the presence of 5 and 20 μg/mL of mChgb. The control cultures did not contain hChgb. Quantifications of the cell count in the primary (C) OPCs and (E) OLs under the condition with 5 and 20 μg/mL of hChgb. The numbers of independent experiments (biological replicates) are 3 in C and 5 in E. The statistical analyses were carried out as described in Materials and methods (**: *p* < 0.01). Error bars represent mean ± s.d.

Although mouse and human Chgbs share the same protein length (677 amino acids), sequence alignment reveals notable different regions. Specifically, mouse Chgb contains a unique repeat of glycine-glutamate-glutamate (QEGEEGEEGEEG, aa. 261-272), whereas human Chgb possesses a distinct region (APEDLEWER, aa. 353-361), absent in the mouse ortholog (S5 Fig). Furthermore, the number of predicted α-helixes differs between mouse and human Chgbs (S5 Fig). Given these variations and the translational relevance of human Chgb to MS pathogenesis, we aimed to validate whether the cytotoxic effects observed with mChgb are also observed with the human protein. We successfully purified hChgb (Fig 3A; S3C, S3D Fig). Interestingly, despite having an identical amino acid number, hChgb showed a higher apparent molecular weight (~100–110 kDa), compared to mChgb (~90 kDa), on SDS-PAGE (Figs 1A and 3A), suggesting differences in post-translational modifications or folding properties driven by sequence divergence. To evaluate cytotoxicity, we treated OPC and OL cultures with hChgb. Consistent with our mChgb findings, hChgb treatment significantly reduced the number of OPCs at 20 μg/mL (Fig 3B, 3C) and the number of differentiated OLs at both 5 μg/mL or 20 μg/mL (Fig 3D, 3E).

**Fig 4:**
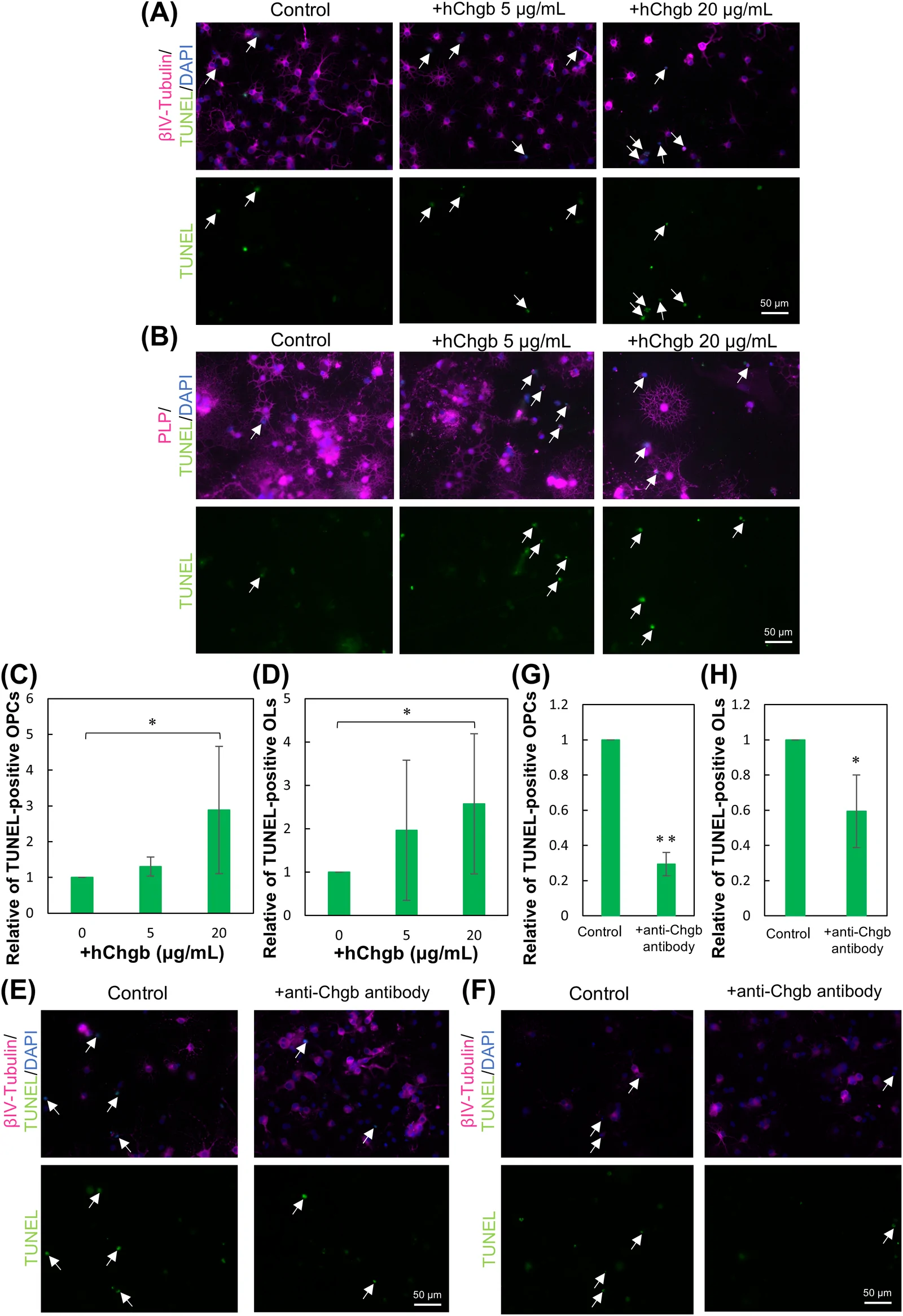
Induction of apoptosis of OPCs and OLs with hChgb. Representative images in TUNEL assays (green) combined with immunostaining of (A) βIV-tubulin (magenta) and (B) PLP (magenta) with DAPI staining (blue) in the primary (A) OPCs and (B) OLs, respectively, in the presence of 5 and 20 μg/mL of hChgb. The controls did not contain hChgb. Arrow: TUNEL-positive OPC or OL. Quantification of TUNEL-positive cell numbers in the primary (C) OPCs and (D) OLs under the condition with 5 and 20 μg/mL of hChgb. The numbers of independent experiments (biological replicates) are 6 in C and 11 in D. Representative images in TUNEL assay (green) combined with immunostaining of βIV-tubulin (magenta) and with DAPI staining (blue) in the primary (E) OPCs and (F) OLs in the presence of 10 μg/mL of hChgb and 10 μg/mL of the anti-Chgb antibody. The controls did not contain the anti-Chgb antibody. Arrow: TUNEL-positive OPC or OL. Quantification of TUNEL-positive cell numbers in the primary (G) OPCs and (H) OLs under the condition with 10 μg/mL of hChgb and 10 μg/mL of the anti-Chgb antibody. The numbers of independent experiments (biological replicates) are 3 in G and 4 in H. The statistical analyses were carried out as described in Materials and methods (*: *p* < 0.05; **: *p* < 0.01). Error bars represent mean ± s.d.

We further confirmed the induction of apoptosis of OPCs and OLs with hChgb by TUNEL assay, and observed that the relative numbers of TUNEL-positive OPCs and OLs to total cells increased at 20 μg/mL hChgb (Fig 4A–4D). Finally, we assessed the effect of the anti-Chgb antibody against the human protein. The antibody effectively inhibited hChgb-induced apoptosis in both cell types (Fig 4E–4H). These results demonstrated that, despite sequence and biochemical differences, the specific pro-apoptotic activity of Chgb toward OL lineage cells is evolutionarily conserved, and that the anti-Chgb neutralizing antibody is effective against both mouse and human Chgbs.

### Endogenous Chgb secreted by neurons triggers OL apoptosis

**Fig 5:**
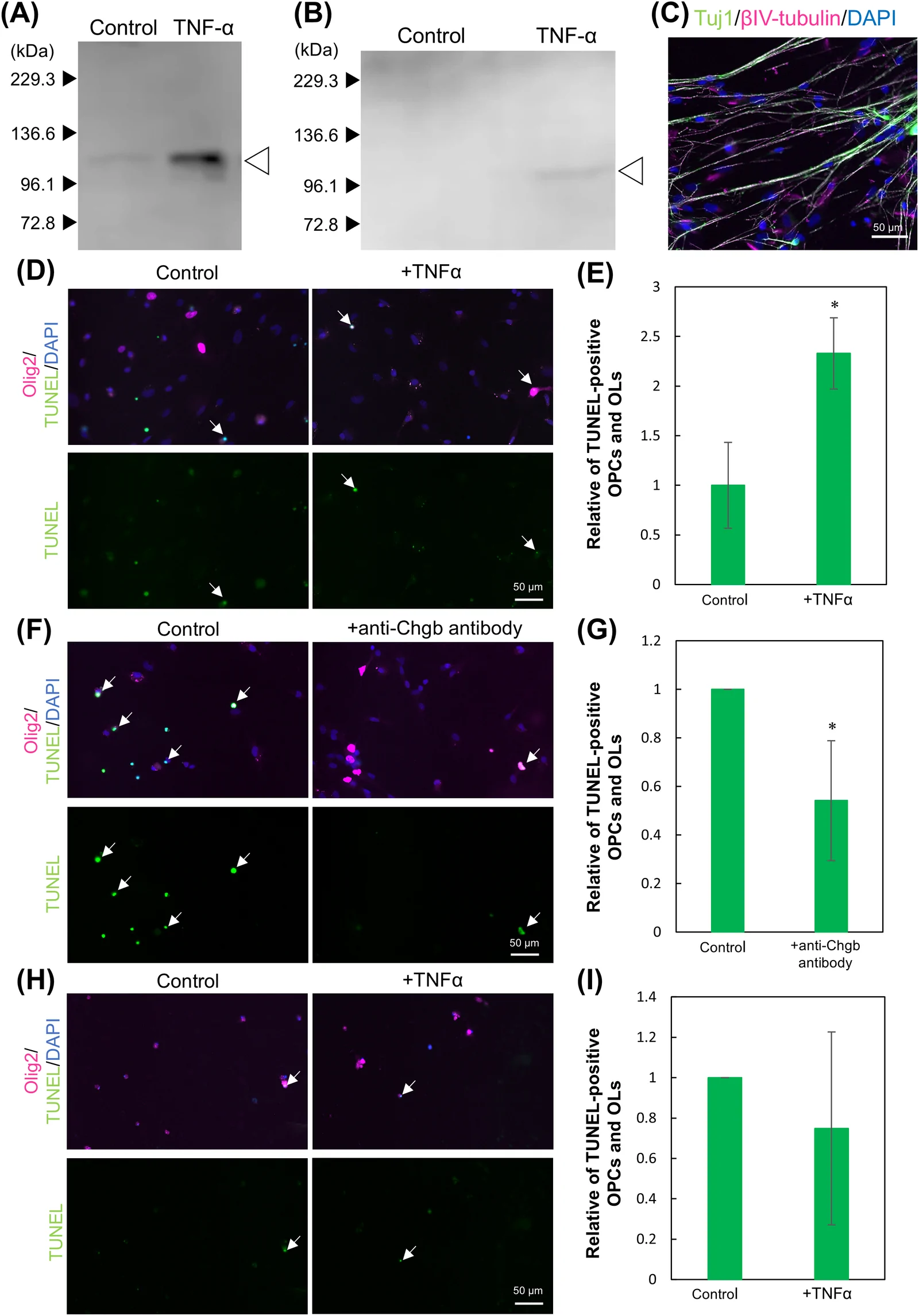
Induction of apoptosis of OLs with endogenous Chgb secreted from neuron in the coculture. (A) Result of western blotting to detect Chgb protein in cell lysates of DRG neurons in the presence of 0.02 μg/mL TNF-α. The control was prepared in the absence of TNF-α. Arrowhead: an expected size of Chgb protein. (B) Result of Western blotting to detect Chgb protein in the conditioned medium of DRG neurons in the presence of 0.02 μg/mL TNF-α. The control was prepared in the absence of TNF-α. Arrowhead: an expected size of Chgb protein. (C) Representative immunostaining images of βIV-tubulin (magenta) and Tuj1(green) with DAPI staining (blue) in the primary coculture of OLs and DRG neurons. (D) Representative images in TUNEL assay (green) combined with immunostaining of Olig2 (magenta) and with DAPI staining (blue) in the primary coculture of OLs and DRG neurons, in the presence of 0.5 μg/mL of TNF-α. The controls did not contain TNF-α. Arrow: TUNEL-positive OL. (E) Quantification of TUNEL-positive OLs numbers in the coculture under the condition with 0.5 μg/mL of TNF-α. The numbers of independent experiments (biological replicates) are 5. (F) Representative images in TUNEL assay (green) combined with immunostaining of Olig2 (magenta) and with DAPI staining (blue) in the primary coculture in the presence of 0.5 μg/mL of TNF-α and 10 μg/mL of the anti-Chgb antibody. The controls did not contain the anti-Chgb antibody. Arrow: TUNEL-positive OL. (G) Quantification of TUNEL-positive OLs numbers in the coculture under the condition with 0.5 μg/mL of TNF-α and 10 μg/mL of the anti-Chgb antibody. The numbers of independent experiments (biological replicates) are 5. (H) Representative images in TUNEL assay (green) combined with immunostaining of Olig2 (magenta) and with DAPI staining (blue) in the primary OLs, in the presence of 0.5 μg/mL of TNF-α. The controls did not contain TNF-α. Arrow: TUNEL-positive OL. (I) Quantification of TUNEL-positive OLs numbers in coculture under the condition with 0.5 μg/mL of TNF-α. The numbers of independent experiments (biological replicates) are 3. The statistical analyses were carried out as described in Materials and methods (*: *p* < 0.05). Error bars represent mean ± s.d.

Finally, we investigated whether endogenous Chgb secreted from neurons exerts similar cytotoxic effects on OPCs and OLs. Based on previous reports that tumor necrosis factor (TNF)-α upregulates Chgb in SHSY5Y neuroblastoma cell line [11], we first assessed whether primary neurons respond similarly. Western blot analysis showed that TNF-α treatment significantly increased Chgb levels in both the cell lysates and conditioned media of DRG neurons, compared to the untreated control (Fig 5A, 5B; S6 Fig). Next, to examine the effect of neuron-derived Chgb on OPC/OL survival, we established a neuron-OPC/OL coculture system. Immunocytochemistry using Tuj1/βIII-tubulin (neuronal marker) and βIV-tubulin (OL lineage marker) confirmed that OL lineage cells were closely associated to neurites within the coculture (Fig 5C). Immunostaining of CNP, a marker for OLs, showed that there were differentiated OLs in this coculture (S7 Fig). We then performed TUNEL assays combined with immunostaining for Olig2, a transcription factor in OL lineage cells, which facilitated the precise quantification of OLs in the mixed culture. Upon TNF-α stimulation to induce Chgb secretion, the relative number of TUNEL-positive OLs significantly increased (Fig 5D, 5E). Importantly, the addition of the neutralizing anti-Chgb antibody suppressed this TNF-α-induced apoptosis (Fig 5F, 5G). To rule out the possibility of direct cytokine toxicity, we confirmed that TNF-α treatment alone did not induce apoptosis in OL monoculture (Fig 5H, 5I). Immunostaining of PLP indicated that there was no significant effect of the TNF-α treatment on OL morphology in the OL monoculture condition (S8 Fig). These results demonstrate that endogenous Chgb secreted by neurons triggers OL apoptosis, mimicking the effects observed with recombinant proteins. Taken together, our findings identify Chgb as a critical neuronal paracrine factor that promotes the apoptotic death of OL lineage cells.

## Discussion

**Fig 6:**
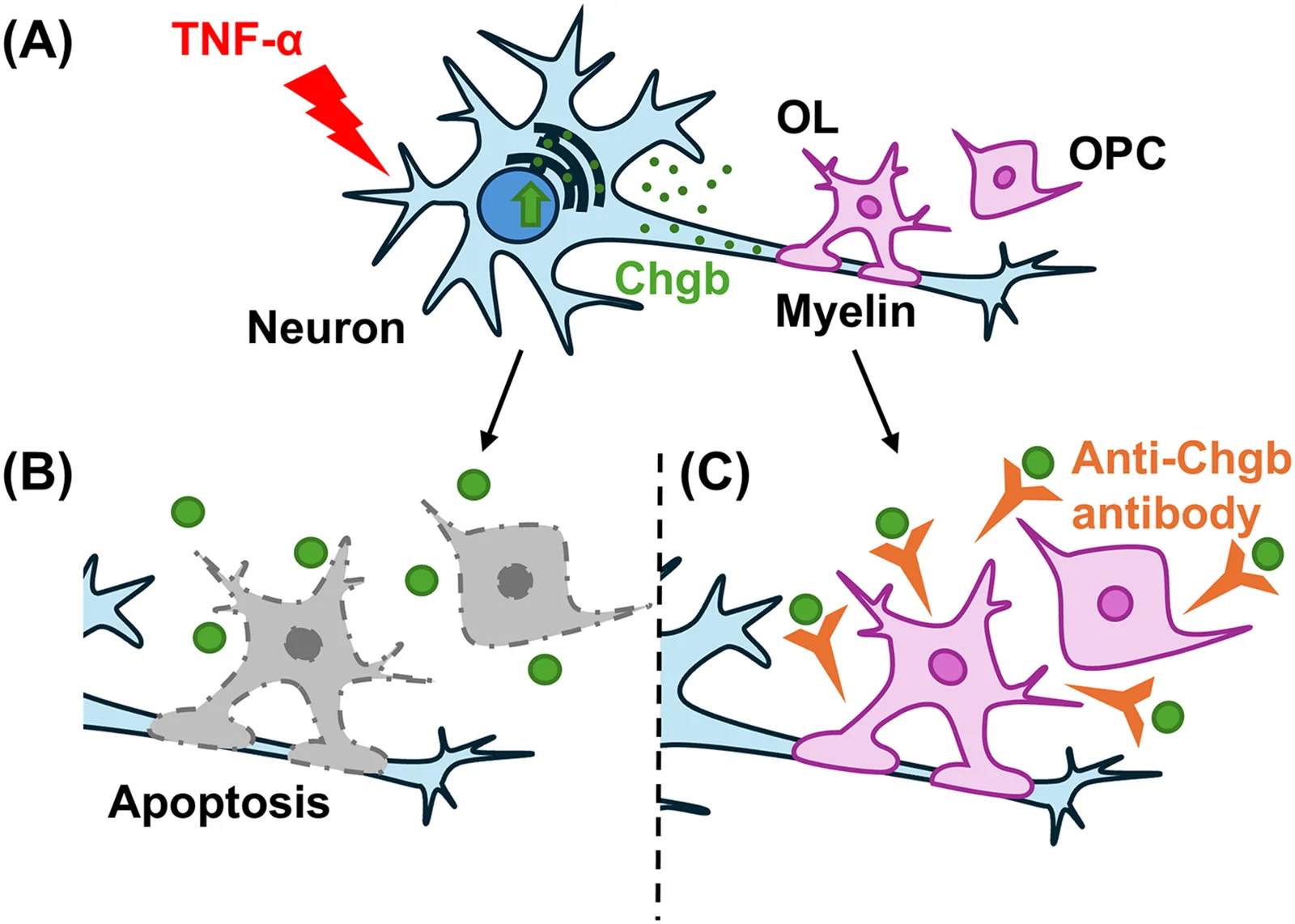
The role of extracellularly secreted Chgb. (A) TNF-α increases the expression level of Chgb in neuron. (B) Chgb secreted from neurons induces apoptosis in OLs and OPCs. (C) The apoptosis is inhibited by anti-Chgb antibody.

In this study, we aimed to reveal the function of extracellular Chgb. We found that the recombinant proteins of both mouse and human Chgbs, regardless of sequence divergence, reduced the number of either OPCs or OLs in each monoculture. In the cultures, TUNEL-positive cells and cleaved caspase-3-positive cells were increased in the presence of recombinant Chgbs. In addition, this OPC/OL apoptosis was blocked by the anti-Chgb antibody. From these results, Chgb possesses the pro-apoptotic activity in OPCs and OLs in their monocultures. Further, we examined the effect of endogenous Chgb secreted from neurons, which was induced by TNF-α, in the coculture of OPCs/OLs and DRG neurons. We observed the increase of TUNEL-positive OPCs/OLs, which was canceled by the anti-Chgb antibody. These results indicate that neuron-derived extracellular Chgb also induces apoptosis of OPCs/OLs in the coculture (Fig 6). To our knowledge, few neuronal soluble factors have been identified as promoters of OPC/OL death; conversely, most known neuronal factors provide positive signals for survival and myelination of these cells [1,13].

A previous study reported that the expression of Chgb is reduced in cerebrospinal fluid of MS patients [10]. A similar decline in extracellular Chgb is observed at the later symptomatic phase in EAE mice, whereas its levels are elevated at the disease onset [11]. At the later stage, the neuronal secretion of Chgb is impaired, resulting in decreased extracellular level [11]. This is consistent with the lower Chgb expression observed in cerebrospinal fluid of MS patients. Mo et al. detected the relative expression level of Chgb protein by Western blotting and immunohistochemistry, compared with the controls, and did not measure the concentration in the tissues [11]. Mattsoon et al. measured the concentrations of fragmental peptides of Chgb in the cerebrospinal fluid of MS patients, but not the whole protein in the CNS tissue, in which OPCs and OLs exist [10]. Thus, the physiological concentration of Chgb in the tissue, particularly at the onset of the symptomatic stage, is not known. One of the limitations in the present study was whether the concentrations of Chgb proteins we tested were appropriate to analyze their activities in the physiological conditions.

In the brain, high Chgb expression is observed in the hippocampus, hypothalamus, amygdala, and cortical layers [14,15]. Dysfunction in these regions is associated with Alzheimer’s disease (AD) and mental disorders, which have recently been characterized as myelin-related diseases [16–18]. In AD, 10–25% of amyloid plaques are Chgb-immunopositive [19]. Furthermore, genetic polymorphisms in Chgb have been identified in Japanese patients with schizophrenia [20]. Moreover, patients with schizophrenia exhibit lower Chgb levels at the chronic stage, contrasting with a rapid increase during the acute stage [21]. These fluctuations in Chgb expression may be caused by mechanisms similar to those observed in MS [10,11].

Our experimental results showed that the anti-Chgb antibody effectively blocked OLs and OPCs apoptosis induced by either human or rodent Chgbs (Fig 6C). In future studies, testing the functional neutralizing antibodies or their derivatives (e.g., single-domain antibodies against Chgb) in EAE or another animal model for demyelination, including the demyelination experiments using Chgb knockout mice will be essential to understand the mechanism of action of these antibodies. Without these *in vivo* experiments, we are unable to properly evaluate the significance of Chgb’s effect on the tissue environment. Particularly, there are various activated immune cells that express inflammatory cytokines and autoantibodies in the demyelinating lesions. For instance, TNF-α induces the expression and secretion of not only Chgb from neurons but also several interleukins and glutamate from microglia and/or astrocytes, directly or indirectly [22–25]. These interleukins and glutamate often reduce the viability and differentiation of OPCs and OLs [26,27]. The effect of Chgb may be masked or cross-talked with the cytokine/glutamate-signaling in the complex *in vivo* environment. In contrast, Chgb clearly induced the apoptotic death of OL lineage cells in the OPC/OL monoculture and the OPC/OL-DRG neurons coculture in this study. Another limitation in this study is that Schwann cells might be contaminated by DRGs in the coculture condition, although we used the well-established method [28,29]. Further experiments to verify these debatable points will help us understand the role of Chgb under physiological conditions.

In the CNS tissue, OLs communicate with not only neurons but also other glial cells for maintaining the homeostasis of the CNS. Gap junctions through connexins are one of the molecular structures for the neuro-glial or interglial communications. Connexins 47 and 43 expressed in OLs and astrocytes, respectively, are critically involved in the regulation of their functions, including the maintenance of myelin formation by OLs [30]. Also, connexin 29 is expressed on OL plasma membrane in the myelin internode region that faces the axonal surface [31]. The activity of Chgb on OL lineage cells may play a role in regulating the homeostatic balances between the CNS cells through these intercellular communications.

In summary, our findings demonstrate that Chgb released from neurons induces apoptosis in the OL lineage cells responsible for CNS myelination. Our findings will help better understanding of OPC/OL biology and cellular relationships between OL lineage cells and neurons.

## Supporting information

S1 Fig The timeline of the culture experiments and immunocytochemical images of differentiation markers. (A) The timeline of the culture experiments for OPCs and OLs is shown. (B) Representative immunostaining images of rabbit anti-NG2 (green) and mouse anti-GalC (magenta) with DAPI staining (blue) in the absence of Chgb. At DIV2, most of the cells were positive for NG2 with their bipolar morphology, which is typical for OPCs, while some cells were positive for GalC in their somas but not processes. At DIV4 of the OPC condition, most cells were still NG2-positive, although their morphology tended to change from bipolar to multipolar, which is typically observed in OPC culture on DIV4. At DIV5 of the OL condition, the majority of the cell populations was positive for GalC, but not for NG2. These GalC-positive cells formed the typical OL morphology with the highly branched cellular processes.
(TIF)

S2 Fig Immunocytochemical analysis of differentiation markers. Representative immunostaining images of (A) rabbit anti-NG2 (green) and mouse anti-βIV-tubulin (magenta) with DAPI staining (blue), (B) rabbit anti-NG2 (green) and mouse anti-NG2 (magenta) with DAPI staining (blue), and (C) rabbit anti-NG2 (green) and rat anti-PDGFRα (magenta) with DAPI staining (blue) at DIV4 in the culture condition for OPCs, and (D) mouse anti-βIV-tubulin (green) and rabbit anti-PLP (magenta) with DAPI staining (blue) at DIV5 in the culture condition for OLs are shown. Cell permeability with 0.1% Triton-X100 was performed in these experiments. Among the OPC markers detected by antibodies raised in mouse or rat, βIV-tubulin was most obviously co-expressed with NG2 in the conditions for OPCs. βIV-tubulin was also co-expressed with PLP in the conditions for OLs.
(TIF)

S3 Fig Whole gel or membrane images of CBB staining and Western blotting analyses of Figs 1A and 3A. Whole images of (A) Fig 1A: CBB staining, (B) Fig 1A: Western blotting, (C) Fig 3A: CBB staining, (D) Fig 3A: Western blotting are shown. Arrow: a lane in which the sample was loaded.
(TIF)

S4 Fig Increase of cleaved caspase-3-positive OPCs and OLs with mChgb. Representative immunostaining images of (A) NG2 (magenta) and cleaved caspase-3 (CC3, green) with DAPI staining (blue) in the culture condition for OPCs and (B) PLP (magenta) and CC3 (green) with DAPI staining (blue) in the culture condition for OLs are shown. In the presence of mChgb, the number of CC3-positive cells increased. Arrow: CC3-positive OPC or OL.
(TIF)

S5 Fig The alignment of amino acid sequences from mouse and human Chgbs (Accession No. mouse: CAA37199.1; human: KAI4004730.1). Multiple Alignment of BLAST at the National Center for Biotechnology Information (NCBI) was used for the alignment. The specific amino acids of either mChgb or hChgb are shown in red and α-helixes are highlighted in light blue. Protein Data bank Japan was used for the prediction of α-helixes (https://pdbj.org/?lang=ja) (Kinjo et al., 2017: https://doi.org/10.1093/nar/gkw962; Kinjo et al., 2018: https://doi.org/10.1002/pro.3273; Bekker et al., 2022: https://doi.org/10.1002/pro.4211).
(TIF)

S6 Fig Whole membrane image of Western blotting analyses of Fig 5A and 5B. Half parenthesis: lanes in which the sample were loaded.
(TIF)

S7 Fig Immunocytochemical images of the coculture of OL lineage cells and DRG neurons. Representative immunostaining image of CNP (magenta) and Tuj1(green) with DAPI staining (blue) in the coculture is shown.
(TIF)

S8 Fig No effect of TGF-α on OLs in the monoculture of OL lineage cells. Representative immunostaining images of PLP (magenta) with DAPI staining (blue) in the presence of 0.5 μg/mL of TNF-α are shown. The control did not contain TNF-α.
(TIF)
