# Supplementary material for: Chromogranin B acts as a neuronal paracrine factor to trigger oligodendrocyte apoptosis
Source: PLoS One. 2026 Sep 21;21(9):e0358362. doi: 10.1371/journal.pone.0358362 (PMC13592581; doi:10.1371/journal.pone.0358362)
Supplement: Supplementary file 1 [file pone.0358362.s010.docx]

**Response to Reviewer #1:**

Thank you very much for your valuable comments. Below is our point-by-point response. Revised parts in the text are highlighted in yellow.

*Reviewer #1: This manuscript investigates the role of neuron-derived Chromogranin B (Chgb) in regulating oligodendrocyte survival and proposes that Chgb acts as a paracrine factor inducing apoptosis in oligodendrocyte lineage cells. Using primary rat OPC and OL cultures, recombinant mouse and human Chgb proteins, and neuron-oligodendrocyte coculture systems, the authors show that extracellular Chgb reduces oligodendrocyte numbers by activating apoptotic pathways, as demonstrated by TUNEL and cleaved caspase-3 assays. They further demonstrate that this effect can be blocked using a neutralizing anti-Chgb antibody, supporting the specificity of the response. The study extends these findings to neuron-derived endogenous Chgb following TNF-α stimulation and links the observations to demyelinating conditions such as multiple sclerosis. Overall, the work provides experimental evidence for a previously underexplored neuron-to-oligodendrocyte death signaling mechanism and offers a potential therapeutic angle.

Points to improve the manuscript:
1. The authors use relatively high concentrations of recombinant Chgb (5–20 μg/mL) in most in vitro experiments. While these doses clearly induce apoptosis, it is not discussed whether these levels are physiologically relevant to what is observed in vivo or in patient CSF. Since the manuscript cites altered Chgb levels in MS and EAE models, it would strengthen the study to either estimate in vivo concentrations or discuss this limitation more explicitly.*

Thank you for your comment. The reference cited in the manuscript detected fragmental peptides of Chgb in the CSF from MS patients by a radioimmunoassay using two different antibodies (antibody CgB 312-331: 2.55 nmol/L; antibody CgB 439-451: 12.45 nmol/L) (Mattsson et al, 2007). These concentrations are lower than those of the recombinant Chgbs we used in this study (mChgb: 56 nM, 110 nM, and 220 nM; hChgb: 48 nM, 95 nM, and 190 nM). However, Mattsson et al. detected the peptides of Chgb in the CSF, but not the whole protein in the tissue where OPCs/OLs exist. Further, at the symptomatic stage, the Chgb expression level is reduced as reported. The other literature did not measure concentrations of Chgb in EAE mouse tissues. They detected Chgb by Western blotting and immunohistochemistry and indicated the relative expression levels of Chgb, compared with the controls (Mo et al, 2013). Thus, the concentration of Chgb in the physiological condition, especially, at the onset of the symptoms, is not known. However, we agree that the point you mentioned is important to discuss. We have added to sentences about this point in Discussion (Page 27, Line 9-17).

*2. The Discussion proposes that extracellular Chgb may act through plasma membrane IP3 receptors to induce calcium overload. However, this remains speculative, and no functional calcium imaging or receptor-blocking experiments are provided. Given that the central claim is that Chgb actively triggers apoptosis, even basic mechanistic validation (for example, calcium measurements or IP3R inhibition) would substantially strengthen the manuscript.*

Thank you for your suggestion. We performed additional experiments to test the effect of an inhibitor of IP3Rs on the apoptotic death of both OPCs and OLs, in the presence of either mChgb or hChgb. As a result, we observed increased apoptosis of both OPCs and OLs by the inhibitor in either case, whereas there were no statistical significances (Additional Data 1). These results do not support our hypothesis. One of the interpretations for the results is that the inhibitor may have predominantly inhibited the function of IP3Rs on ER membrane, since the majority of IP3Rs is expressed on ER membrane, but not on plasma membrane. Or a different protein on plasma membrane functions as a receptor for Chgb. In any case, we cannot emphasize the hypothesis. We have toned down to explain the hypothesis in Discussion (Page 26, Line 11-13).

*3. All functional data are derived from primary cultures and coculture models. While these systems are well established and carefully executed, the lack of in vivo validation limits the translational impact. Given the strong disease relevance proposed, even limited in vivo confirmation (e.g., antibody treatment in an EAE model or tissue-level apoptosis analysis) would greatly enhance confidence in the conclusions.*

We agreed that it is a very important point. However, it takes much time to set up these *in vivo* or *ex vivo* experiments and it was impossible to obtain a promising result during this revision period. We will try to perform these experiments in our next study. Instead, we have described this point as a limitation of this study and have toned down to explain the physiological role of Chgb in Discussion (Page 28, Line 8-17).

*4. The authors show that TNF-α induces Chgb secretion from neurons and indirectly promotes oligodendrocyte apoptosis. Although they control for direct TNF-α toxicity in monoculture, TNF-α is a complex cytokine with multiple downstream effects, including the induction of other cytokines such as IL-6, IL-1β, IL-10, and TGF-β. A more detailed discussion of possible indirect pathways, beyond Chgb alone, would make the interpretation more balanced.*

Thank you for your comment and advice. We have added descriptions of the effects of the cytokines on Chgb activity in Discussion (Page 28, Line 12-17).

*5. The manuscript repeatedly suggests that anti-Chgb antibodies may represent a promising therapeutic strategy. While the in vitro neutralization data are convincing, the absence of in vivo efficacy and safety data makes this claim somewhat speculative. The authors may wish to tone down this aspect or frame it more clearly as a long-term possibility rather than an immediate translational outcome.*

We agreed with your opinion. We have toned down to explain the possibility of anti-Chgb antibodies as therapeutic agents in the manuscript (Page 2, Line 16; Page 6, Line 4; Page 28, Line 8-9; Page 29, Line 6-8).

*6. Several of the immunofluorescence panels, particularly in the main figures and supplementary data, are not consistently presented at a high technical standard. In multiple cases, the signal appears either weak or uneven, and in some images, the fluorescence seems oversaturated, potentially masking cellular details and affecting interpretability. Since these images form a major basis for the quantitative analyses, improving image acquisition and presentation (including appropriate exposure settings and dynamic range) would strengthen confidence in the data. Where possible, providing representative low- and high-magnification views and avoiding overexposed regions would further enhance the clarity and reproducibility of the findings.*

Thank you for your comment. We have replaced and added several images in main and supplementary figures. In particular, we carefully selected the images that exhibit cellular morphology as clearly as possible, except for the images with nuclear markers, such as TUNEL and Olig2 staining (Figs. 1B, D, 3B, D, S1B, S2B, C, S7, and S8).

*7. The authors could also highlight another angle of the scenario involving the gap junction intercellular communication proteins between neurons and oligodendrocytes. Recent advances suggest that astrocytes and oligodendrocytes interact with the expression of specific gap junction proteins, such as Cx43 and Cx47, respectively. These junctional proteins can be crucial for transmitting specific signals and for influencing the overall homeostasis of the CNS. This may be currently out of scope for the authors, but they could consider mentioning it as a future perspective in the discussion section. This will enhance the manuscript by highlighting another angle of transmission of Chgb within cells.*

Thank you for the interesting information. We have added a speculative mechanism of Chgb functions in the intercellular networks through connexins in Discussion (Page 26, Line 15-18; Page 27, Line 1-4).

*Minor Comments
1. The statistical section states that experiments were performed “at least 3 times,” but exact biological replicate numbers are not always clear in the figure legends. Providing precise n values for both technical and biological replicates would improve transparency.*

Thank you for your advice. We have added to describe the number of the independent experiments (biological replicates) in each Figure legend (Page 16, Line 17-18; Page 18, Line 9-10; Page 18, Line 15-16; Page 20, Line 5-6; Page 21, Line 5-6; Page 21, Line 11-12; Page 23, Line 16; Page 24, Line 3-4; Page 24, Line 8-9). There is a variability in the number of independent experiments between the analyses, because in some of the experiments, all the prepared cells, including cells for controls, were already more differentiated when plated, or were less differentiated after induction of differentiation. These were probably due to the different timing of their differentiation between the independent experiments, while they were cultured in the mixed glial culture from different rat cerebral cortexes. The number of technical replicates was one in all the independent experiments since the cell number in each independent experiment from the mixed glial culture was not enough to prepare multiple technical replicants for all the experimental conditions. We have added this explanation in Materials and Methods (Page 15, Line 1-8).

*2. There are minor typographical and formatting inconsistencies (for example, spacing around symbols and occasional line-break artifacts) that should be corrected during revision.*

Thank you for letting us know. We have carefully corrected these parts throughout the text.

*3. The Discussion could benefit from a clearer separation between experimentally supported conclusions and speculative hypotheses.*

Thank you for your suggestion. However, it was difficult to clearly separate them, although we tried to do it as much as possible. We have described the experimentally supported conclusion in the first paragraph. The second and third paragraphs are more speculatively hypothetic sections of cellular and molecular mechanisms. The fourth paragraph contains the physiological relevance of Chgb in diseases and a limitation regarding its concentration as you mentioned above. In the fifth paragraph, we have described discussions about the cytokine effects and limitations of this study as you and Reviewer #2 suggested to discuss. The sixth paragraph is the summary section. We hope this shape of the Discussion sections would be accepted.

**Response to Reviewer #2:**

Thank you very much for your valuable comments. Below is our point-by-point response. Revised parts in the text are highlighted in yellow.

*Reviewer #2: In this study, the authors investigated the effect of extracellular chromogranin B (Chgb) on oligodendrocyte lineage cells. The study aimed to explore and discuss the role of neuron-derived ChGB in remyelination in multiple sclerosis. While the study raises some interesting points, the experimental design and the results are unclear in many respects, which undermines the soundness of the study and the interpretation of the result*

*Major points

1. Line 207: 'OPCs were cultured in OPC medium for three days, and OLs were cultured in OL medium for three days'. Please provide a timeline indicating the complete process of culture preparation, including the stage-specific markers used in the study, as well as micrographs of the expected morphology. Please declare the DIV in each experiment.*

Thank you for your suggestion. We have prepared a supplementary figure (S1 Fig), which indicates the timeline of the culture experiments and images of OPCs and OLs with their specific markers, NG2 and GalC, respectively, in the absence of Chgb. At DIV2, most of the cells were positive for NG2 with their bipolar morphology, which is typical for OPCs, while some cells were positive for GalC in their somas but not processes. At DIV4 of the OPC condition, most cells were still NG2-positive, although their morphology tended to change from bipolar to multipolar, which is typically observed in OPC culture on DIV4. At DIV5 of the OL condition, the majority in the cell populations was positive for GalC, but not for NG2. These GalC-positive cells formed the typical OL morphology with the highly branched cellular processes. We have added descriptions about this data in Materials and Methods (Page 10, Line 5-7).

*2. The use of βIV-tubulin as an OPC marker expressed throughout the oligo lineage is highly questionable. Why not use NG2 or other markers that are clearly associated with the undifferentiated, proliferative stage?*

In this study, we needed to perform the detergent treatment for cell permeability in TUNEL assay. In addition, we added the rabbit anti-Chgb antibody in the cultures for the inhibition assay. Therefore, it was necessary to use marker antibodies, which were detergent-resident and raised except in rabbit. We tested three antibodies to find the one that works well for the detection of OPCs in this specific condition. As a result, mouse anti-βIV-tubulin antibody was most suitable among them. The mouse anti-βIV-tubulin antibody also detected OLs in the condition as well. We have added images in S2 Fig and have added a description in Materials and Methods (Page 13, Line 16-18; Page 14, Line 1-2).

*3. In the figures, PLP-positive cells exhibit different morphological phenotypes: quite immature in Fig. 1 and Fig. 3, and convincing as OLs in Fig. 4. Are the DIVs the same? If so, could the authors provide different micrographs or explain this difference?*

Thank you for pointing it. We have replaced images of PLP-positive cells in Figs. 1D and 3D. These images were taken on DIV5 of the OL culture condition (see in S1 Fig), same as the images in Fig. 4B.

*4. In the experimental design, the toxic effect of Chgb is tested under standard culture conditions. Since all the results are discussed in terms of inflammatory-demyelinating diseases and cells of the oligodendrocyte lineage are very sensitive to inflammatory cytokines in vitro, the study would benefit from investigating the effect of Chgb in the presence of inflammatory challenges and TNFα.*

Thank you for your comment. We agreed that it is an important point. The expression of the cytokines, such as several interleukins, which are induced by TNF-α, is observed in astrocytes, microglia, and other activated immune cells. We are currently planning to set up *in vivo* and *ex vivo* experiments to analyze the effect of Chgb in the complex condition with the expression of the various inflammatory cytokines, as you pointed. However, it takes time to set up these experiments and it was impossible to obtain a convincing result during this revision period. We will try to perform these experiments in our next study. Instead, we have described these points as a limitation of this study and have toned down to explain the physiological role of Chgb in Discussion (Page 28, Line 8-17).

*5. Co-culture: the authors used OPC/OL derived from the cerebral cortex and DRG-derived neurons. Although this is a very popular in vitro myelination assay, it is highly questionable given that Schwann cells are the myelinating cells of DRG axons. This must be discussed as a limitation of the study.*

Thank you for pointing it. We have added to a description of the limitation in Discussion (Page 28, Line 18; Page 29, Line 1)*.*

*6. Figure 5 and co-culture experiment: the cells presented in Figure 5 seem to be OPCs, not OLs (as stated in line 209). This is because Olig2 is a transcription factor that regulates the very early stages of the oligodendrocyte lineage, and the morphology is consistent with that of OPCs. How long were the cells cultured for? This is a key point since OPCs and OLs play different roles in inflammatory-demyelinating conditions, albeit coherent ones. In this experiment, the author states that TNFα does not induce apoptosis in OL monocultures. This is quite surprising, given that extensive literature indicates TNFα as a major factor in OL degeneration in inflammatory demyelinating diseases and in vitro models.*

Thank you for the important comment. We cocultured these cells for 5 days. At the time point we analyzed, we observed many CNP-positive OLs, whose morphology was typically OL-like. We have prepared a supplementary figure to show it (S7 Fig) and have added a description (Page 22, Line 8-9). The reason we used anti-Olig2 antibody was that it worked well for counting nuclei of OL lineage cells in the coculture condition. Staining of either βIV-tubulin or CNP was relatively difficult to count OL lineage cells due to the weak non-specific signal on DRG neurons. As you mentioned, Olig2 expression is intense during OPC stage, but it lasts at the moderate level after differentiation and is used as a marker for OLs as well as OPCs (Nishiyama et al, 2009, *Nat Rev Neurosci*). Thus, we have revised descriptions of only “OLs” to “OPCs/OLs” or “OL lineage cells” regarding this coculture experiment throughout the entire text.

In terms of the effect of TNF-α on OLs, we could not observe a significant increase in apoptotic cell death in their monoculture with TNF-α, even though its concentration was relatively high (500 ng/mL). We additionally performed immunostaining of PLP in the same condition, and the result exhibited the fine OL morphology (S8 Fig) (Page 22, Line 15-17). In some literature, no significant induction of OL lineage cell death by TNF-α is reported, depending on its concentration (100 ng/mL: they did not test higher concentrations), whereas the others clearly show their apoptotic death with the same concentration (Kim et al, 2011, *J Neurochem*; Bonora et al, 2014, *Cell Death Differen*). This discrepancy may be due to differences in culture conditions and the purity/activity of different commercial products/lots of TNF-α. In our coculture experiment, the anti-Chgb antibody significantly blocked apoptosis of OL lineage cells, indicating that the main inducer of apoptosis is Chgb in this condition. However, we agreed that TNF-α and other cytokines negatively influence on OPC/OL survival. Therefore, we have described these cytokine effects in Discussion (Page 28, Line 12-17).
