# Supplementary material for: Chromogranin B acts as a neuronal paracrine factor to trigger oligodendrocyte apoptosis
Source: PLoS One. 2026 Sep 21;21(9):e0358362. doi: 10.1371/journal.pone.0358362 (PMC13592581; doi:10.1371/journal.pone.0358362)
Supplement: Supplementary file 2 [file pone.0358362.s011.docx]

**Response to Reviewer #1:**

Thank you very much for your valuable comments. Below is our response. Revised parts in the text are highlighted in yellow.

*Reviewer #1: The revised manuscript is improved, and many of the earlier concerns have been addressed. However, an important issue remains regarding the mechanistic interpretation. The additional IP3R inhibitor experiments were specifically performed to support the proposed mechanism that extracellular Chgb induces apoptosis through IP3R-related signaling. However, the authors themselves state that these experiments did not support the hypothesis, since the inhibitor failed to produce statistically significant effects. This is an important negative result and should be treated more explicitly in the manuscript. At present, the study convincingly shows that recombinant and neuron-derived Chgb can induce apoptosis in OL-lineage cells in vitro, but the mechanistic basis remains unresolved. Similarly, the lack of in vivo or ex vivo validation means the direct relevance to demyelinating disease pathology remains speculative. These limitations do not invalidate the cell culture findings, but they substantially weaken the biological and translational conclusions.

The manuscript should avoid implying that the signaling pathway itself has been established. The absence of in vivo or ex vivo validation becomes more important in light of the unresolved mechanism. At present, the manuscript demonstrates a cell culture phenomenon but has not yet identified a validated pathological pathway in demyelinating disease. The therapeutic discussion regarding anti-Chgb antibodies should remain conservative, as the receptor mechanism and in vivo disease relevance remain unclear. Although several IF panels have improved, some microscopy images still appear uneven or partially oversaturated. A final careful review of image presentation would improve overall figure quality.

I have given a major revision with the understanding that these experiments are crucial for the current manuscript, and I have also submitted a comment to the editor regarding checking the journal policy and taking a stand on whether a major revision is required. If the new in vivo experiments and the other experiments cannot be added, the paper should be rewritten with the available results that align with the hypothesis.*

Thank you for your comment. As we explained in the last revision, it takes much time to set up the *in vivo* or *ex vivo* experiments and it was very difficult to obtain a promising result during this revision period. We will challenge these experiments, including experiments for the elucidation of the molecular mechanism, in our next study. We hope you could understand the situation.

We agree that we should not emphasize speculations about the molecular mechanism and the physiological relevance without experimental data. We have eliminated the paragraphs regarding the molecular mechanism and the possible physiological function from Discussion (2nd and 3rd paragraphs in the former version) (We have kept and moved the part described about gap junction proteins, which you suggested in the former revision: Page 27, Line 10-17). Instead, we have added a paragraph that summarize the available results and conclusion indicated by the results in Discussion (Page 24, Line 11-18; Page 25, Line 1-4). In addition, we have revised several sentences, which meant possible physiological and pathological functions of Chgb in Abstract, Introduction, and Discussion (Page 2, Line 14-15; Page 6, Line 1-2; Page 28, Line 1-3).

In terms of the oversaturated fluorescent images, we have replaced them with better ones (Figs. 1D, 3D, S1B, and S2A). We chose the images that are not oversaturated but show cellular morphology, including branched processes. We hope these images fit with the criteria. Thank you so much for your consideration.

**Response to Reviewer #2:**

*Reviewer #2: All comments have been addressed. I would simply suggest to group the study limitations in a dedicated paragraph*

We appreciate for your review and comment. We will work on the limitations you pointed in our next study.
