# Supplementary material for: Chromogranin B acts as a neuronal paracrine factor to trigger oligodendrocyte apoptosis
Source: PLoS One. 2026 Sep 21;21(9):e0358362. doi: 10.1371/journal.pone.0358362 (PMC13592581; doi:10.1371/journal.pone.0358362)
Supplement: Supplementary file 3 [file pone.0358362.s012.docx]

**Comment from the academic editor:**

Thus, only add the comments in the discussion, like limitations or in future experiments.

**Response to the editor’s comment:**

Thank you very much for your comment. We have revised a paragraph about limitations of this study and future experiments in Discussion (Page 26, Line 14-18; Page 27, Line 7-9). Revised parts in the text are highlighted in yellow.
