# Supplementary material for: Chromogranin B acts as a neuronal paracrine factor to trigger oligodendrocyte apoptosis
Source: PLoS One. 2026 Sep 21;21(9):e0358362. doi: 10.1371/journal.pone.0358362 (PMC13592581; doi:10.1371/journal.pone.0358362)
Supplement: Peer review history

# Peer review: Decision Letter 0

**DOI:** 10.1371/journal.pone.0358362.r001

**Authors:** Eliseo Eugenin

3 Mar 2026

Dear Dr. Suzuki,

Thank you for submitting your manuscript to PLOS ONE. After careful consideration, we feel that it has merit but does not fully meet PLOS ONE’s publication criteria as it currently stands. Therefore, we invite you to submit a revised version of the manuscript that addresses the points raised during the review process.

==============================

Dear Dr. Suzuki

Thank you for submitting your manuscript to PLOSone. Both reviewers had significant suggestions and questions about the manuscript that need your attention. Please answer all the concerns and label the changes in the manuscript.

best regards

Eliseo Eugenin

==============================

Please submit your revised manuscript by Apr 17 2026 11:59PM. If you will need more time than this to complete your revisions, please reply to this message or contact the journal office at plosone@plos.org. When you're ready to submit your revision, log on to https://www.editorialmanager.com/pone/ and select the 'Submissions Needing Revision' folder to locate your manuscript file.

- A letter that responds to each point raised by the academic editor and reviewer(s). You should upload this letter as a separate file labeled 'Response to Reviewers'.
- A marked-up copy of your manuscript that highlights changes made to the original version. You should upload this as a separate file labeled 'Revised Manuscript with Track Changes'.
- An unmarked version of your revised paper without tracked changes. You should upload this as a separate file labeled 'Manuscript'.

If you would like to make changes to your financial disclosure, please include your updated statement in your cover letter. Guidelines for resubmitting your figure files are available below the reviewer comments at the end of this letter.

If applicable, we recommend that you deposit your laboratory protocols in protocols.io to enhance the reproducibility of your results. Protocols.io assigns your protocol its own identifier (DOI) so that it can be cited independently in the future. For instructions see: https://journals.plos.org/plosone/s/submission-guidelines#loc-laboratory-protocols. Additionally, PLOS ONE offers an option for publishing peer-reviewed Lab Protocol articles, which describe protocols hosted on protocols.io. Read more information on sharing protocols at https://plos.org/protocols?utm_medium=editorial-email&utm_source=authorletters&utm_campaign=protocols.

We look forward to receiving your revised manuscript.

Kind regards,

Eliseo A Eugenin, Ph.D.

Academic Editor

PLOS One

Journal Requirements:

When submitting your revision, we need you to address these additional requirements.

1. Please ensure that your manuscript meets PLOS ONE's style requirements, including those for file naming. The PLOS ONE style templates can be found at

https://journals.plos.org/plosone/s/file?id=wjVg/PLOSOne_formatting_sample_main_body.pdf and

https://journals.plos.org/plosone/s/file?id=ba62/PLOSOne_formatting_sample_title_authors_affiliations.pdf

2.To comply with PLOS One submissions requirements, in your Methods section, please provide additional information regarding the experiments involving animals and ensure you have included details on (1) methods of anesthesia and/or analgesia, and (2) efforts to alleviate suffering.

3. PLOS ONE now requires that authors provide the original uncropped and unadjusted images underlying all blot or gel results reported in a submission’s figures or Supporting Information files. This policy and the journal’s other requirements for blot/gel reporting and figure preparation are described in detail at https://journals.plos.org/plosone/s/figures#loc-blot-and-gel-reporting-requirements and https://journals.plos.org/plosone/s/figures#loc-preparing-figures-from-image-files. When you submit your revised manuscript, please ensure that your figures adhere fully to these guidelines and provide the original underlying images for all blot or gel data reported in your submission. See the following link for instructions on providing the original image data: https://journals.plos.org/plosone/s/figures#loc-original-images-for-blots-and-gels.

In your cover letter, please note whether your blot/gel image data are in Supporting Information or posted at a public data repository, provide the repository URL if relevant, and provide specific details as to which raw blot/gel images, if any, are not available. Email us at plosone@plos.org if you have any questions.

4. If the reviewer comments include a recommendation to cite specific previously published works, please review and evaluate these publications to determine whether they are relevant and should be cited. There is no requirement to cite these works unless the editor has indicated otherwise.

Additional Editor Comments:

Dear Dr. Suzuki

Thank you for submitting your manuscript to PLOSone. Both reviewers had significant suggestions and questions about the manuscript that need your attention. Please answer all the concerns and label the changes in the manuscript.

best regards

Eliseo Eugenin

[Note: HTML markup is below. Please do not edit.]

Reviewers' comments:

Reviewer's Responses to Questions

**Comments to the Author**

1. Is the manuscript technically sound, and do the data support the conclusions?

Reviewer #1: Yes

Reviewer #2: No

**********

2. Has the statistical analysis been performed appropriately and rigorously?

Reviewer #1: No

Reviewer #2: Yes

**********

3. Have the authors made all data underlying the findings in their manuscript fully available?

The PLOS Data policy

Reviewer #1: Yes

Reviewer #2: No

**********

4. Is the manuscript presented in an intelligible fashion and written in standard English?

Reviewer #1: Yes

Reviewer #2: Yes

**********

Reviewer #1: This manuscript investigates the role of neuron-derived Chromogranin B (Chgb) in regulating oligodendrocyte survival and proposes that Chgb acts as a paracrine factor inducing apoptosis in oligodendrocyte lineage cells. Using primary rat OPC and OL cultures, recombinant mouse and human Chgb proteins, and neuron-oligodendrocyte coculture systems, the authors show that extracellular Chgb reduces oligodendrocyte numbers by activating apoptotic pathways, as demonstrated by TUNEL and cleaved caspase-3 assays. They further demonstrate that this effect can be blocked using a neutralizing anti-Chgb antibody, supporting the specificity of the response. The study extends these findings to neuron-derived endogenous Chgb following TNF-α stimulation and links the observations to demyelinating conditions such as multiple sclerosis. Overall, the work provides experimental evidence for a previously underexplored neuron-to-oligodendrocyte death signaling mechanism and offers a potential therapeutic angle.

Points to improve the manuscript:

1. The authors use relatively high concentrations of recombinant Chgb (5–20 μg/mL) in most in vitro experiments. While these doses clearly induce apoptosis, it is not discussed whether these levels are physiologically relevant to what is observed in vivo or in patient CSF. Since the manuscript cites altered Chgb levels in MS and EAE models, it would strengthen the study to either estimate in vivo concentrations or discuss this limitation more explicitly.

2. The Discussion proposes that extracellular Chgb may act through plasma membrane IP3 receptors to induce calcium overload. However, this remains speculative, and no functional calcium imaging or receptor-blocking experiments are provided. Given that the central claim is that Chgb actively triggers apoptosis, even basic mechanistic validation (for example, calcium measurements or IP3R inhibition) would substantially strengthen the manuscript.

3. All functional data are derived from primary cultures and coculture models. While these systems are well established and carefully executed, the lack of in vivo validation limits the translational impact. Given the strong disease relevance proposed, even limited in vivo confirmation (e.g., antibody treatment in an EAE model or tissue-level apoptosis analysis) would greatly enhance confidence in the conclusions.

4. The authors show that TNF-α induces Chgb secretion from neurons and indirectly promotes oligodendrocyte apoptosis. Although they control for direct TNF-α toxicity in monoculture, TNF-α is a complex cytokine with multiple downstream effects, including the induction of other cytokines such as IL-6, IL-1β, IL-10, and TGF-β. A more detailed discussion of possible indirect pathways, beyond Chgb alone, would make the interpretation more balanced.

5. The manuscript repeatedly suggests that anti-Chgb antibodies may represent a promising therapeutic strategy. While the in vitro neutralization data are convincing, the absence of in vivo efficacy and safety data makes this claim somewhat speculative. The authors may wish to tone down this aspect or frame it more clearly as a long-term possibility rather than an immediate translational outcome.

6. Several of the immunofluorescence panels, particularly in the main figures and supplementary data, are not consistently presented at a high technical standard. In multiple cases, the signal appears either weak or uneven, and in some images, the fluorescence seems oversaturated, potentially masking cellular details and affecting interpretability. Since these images form a major basis for the quantitative analyses, improving image acquisition and presentation (including appropriate exposure settings and dynamic range) would strengthen confidence in the data. Where possible, providing representative low- and high-magnification views and avoiding overexposed regions would further enhance the clarity and reproducibility of the findings.

7. The authors could also highlight another angle of the scenario involving the gap junction intercellular communication proteins between neurons and oligodendrocytes. Recent advances suggest that astrocytes and oligodendrocytes interact with the expression of specific gap junction proteins, such as Cx43 and Cx47, respectively. These junctional proteins can be crucial for transmitting specific signals and for influencing the overall homeostasis of the CNS. This may be currently out of scope for the authors, but they could consider mentioning it as a future perspective in the discussion section. This will enhance the manuscript by highlighting another angle of transmission of Chgb within cells.

Minor Comments

1. The statistical section states that experiments were performed “at least 3 times,” but exact biological replicate numbers are not always clear in the figure legends. Providing precise n values for both technical and biological replicates would improve transparency.

2. There are minor typographical and formatting inconsistencies (for example, spacing around symbols and occasional line-break artifacts) that should be corrected during revision.

3. The Discussion could benefit from a clearer separation between experimentally supported conclusions and speculative hypotheses.

Reviewer #2: In this study, the authors investigated the effect of extracellular chromogranin B (Chgb) on oligodendrocyte lineage cells. The study aimed to explore and discuss the role of neuron-derived ChGB in remyelination in multiple sclerosis. While the study raises some interesting points, the experimental design and the results are unclear in many respects, which undermines the soundness of the study and the interpretation of the result

Major points

1. Line 207: 'OPCs were cultured in OPC medium for three days, and OLs were cultured in OL medium for three days'. Please provide a timeline indicating the complete process of culture preparation, including the stage-specific markers used in the study, as well as micrographs of the expected morphology. Please declare the DIV in each experiment.

2. The use of βIV-tubulin as an OPC marker expressed throughout the oligo lineage is highly questionable. Why not use NG2 or other markers that are clearly associated with the undifferentiated, proliferative stage?

3. In the figures, PLP-positive cells exhibit different morphological phenotypes: quite immature in Fig. 1 and Fig. 3, and convincing as OLs in Fig. 4. Are the DIVs the same? If so, could the authors provide different micrographs or explain this difference?

4. In the experimental design, the toxic effect of Chgb is tested under standard culture conditions. Since all the results are discussed in terms of inflammatory-demyelinating diseases and cells of the oligodendrocyte lineage are very sensitive to inflammatory cytokines in vitro, the study would benefit from investigating the effect of Chgb in the presence of inflammatory challenges and TNFα.

5. Co-culture: the authors used OPC/OL derived from the cerebral cortex and DRG-derived neurons. Although this is a very popular in vitro myelination assay, it is highly questionable given that Schwann cells are the myelinating cells of DRG axons. This must be discussed as a limitation of the study.

6. Figure 5 and co-culture experiment: the cells presented in Figure 5 seem to be OPCs, not OLs (as stated in line 209). This is because Olig2 is a transcription factor that regulates the very early stages of the oligodendrocyte lineage, and the morphology is consistent with that of OPCs. How long were the cells cultured for? This is a key point since OPCs and OLs play different roles in inflammatory-demyelinating conditions, albeit coherent ones. In this experiment, the author states that TNFα does not induce apoptosis in OL monocultures. This is quite surprising, given that extensive literature indicates TNFα as a major factor in OL degeneration in inflammatory demyelinating diseases and in vitro models.

**********

what does this mean?). If published, this will include your full peer review and any attached files.

If you choose “no”, your identity will remain anonymous but your review may still be made public.

**Do you want your identity to be public for this peer review?**  For information about this choice, including consent withdrawal, please see our Privacy Policy

Reviewer #1: No

Reviewer #2: **Yes:**  Laura Calza

**********

[NOTE: If reviewer comments were submitted as an attachment file, they will be attached to this email and accessible via the submission site. Please log into your account, locate the manuscript record, and check for the action link "View Attachments". If this link does not appear, there are no attachment files.]

To ensure your figures meet our technical requirements, please review our figure guidelines: https://journals.plos.org/plosone/s/figures

You may also use PLOS’s free figure tool, NAAS, to help you prepare publication quality figures: https://journals.plos.org/plosone/s/figures#loc-tools-for-figure-preparation.

NAAS will assess whether your figures meet our technical requirements by comparing each figure against our figure specifications.

# Author comment: Author response to Decision Letter 1

**DOI:** 10.1371/journal.pone.0358362.r002

29 Apr 2026

Response to Reviewer #1:

Thank you very much for your valuable comments. Below is our point-by-point response. Revised parts in the text are highlighted in yellow.

Reviewer #1: This manuscript investigates the role of neuron-derived Chromogranin B (Chgb) in regulating oligodendrocyte survival and proposes that Chgb acts as a paracrine factor inducing apoptosis in oligodendrocyte lineage cells. Using primary rat OPC and OL cultures, recombinant mouse and human Chgb proteins, and neuron-oligodendrocyte coculture systems, the authors show that extracellular Chgb reduces oligodendrocyte numbers by activating apoptotic pathways, as demonstrated by TUNEL and cleaved caspase-3 assays. They further demonstrate that this effect can be blocked using a neutralizing anti-Chgb antibody, supporting the specificity of the response. The study extends these findings to neuron-derived endogenous Chgb following TNF-α stimulation and links the observations to demyelinating conditions such as multiple sclerosis. Overall, the work provides experimental evidence for a previously underexplored neuron-to-oligodendrocyte death signaling mechanism and offers a potential therapeutic angle.

Points to improve the manuscript:

1. The authors use relatively high concentrations of recombinant Chgb (5–20 μg/mL) in most in vitro experiments. While these doses clearly induce apoptosis, it is not discussed whether these levels are physiologically relevant to what is observed in vivo or in patient CSF. Since the manuscript cites altered Chgb levels in MS and EAE models, it would strengthen the study to either estimate in vivo concentrations or discuss this limitation more explicitly.

Thank you for your comment. The reference cited in the manuscript detected fragmental peptides of Chgb in the CSF from MS patients by a radioimmunoassay using two different antibodies (antibody CgB 312-331: 2.55 nmol/L; antibody CgB 439-451: 12.45 nmol/L) (Mattsson et al, 2007). These concentrations are lower than those of the recombinant Chgbs we used in this study (mChgb: 56 nM, 110 nM, and 220 nM; hChgb: 48 nM, 95 nM, and 190 nM). However, Mattsson et al. detected the peptides of Chgb in the CSF, but not the whole protein in the tissue where OPCs/OLs exist. Further, at the symptomatic stage, the Chgb expression level is reduced as reported. The other literature did not measure concentrations of Chgb in EAE mouse tissues. They detected Chgb by Western blotting and immunohistochemistry and indicated the relative expression levels of Chgb, compared with the controls (Mo et al, 2013). Thus, the concentration of Chgb in the physiological condition, especially, at the onset of the symptoms, is not known. However, we agree that the point you mentioned is important to discuss. We have added to sentences about this point in Discussion (Page 27, Line 9-17).

2. The Discussion proposes that extracellular Chgb may act through plasma membrane IP3 receptors to induce calcium overload. However, this remains speculative, and no functional calcium imaging or receptor-blocking experiments are provided. Given that the central claim is that Chgb actively triggers apoptosis, even basic mechanistic validation (for example, calcium measurements or IP3R inhibition) would substantially strengthen the manuscript.

Thank you for your suggestion. We performed additional experiments to test the effect of an inhibitor of IP3Rs on the apoptotic death of both OPCs and OLs, in the presence of either mChgb or hChgb. As a result, we observed increased apoptosis of both OPCs and OLs by the inhibitor in either case, whereas there were no statistical significances (Additional Data 1). These results do not support our hypothesis. One of the interpretations for the results is that the inhibitor may have predominantly inhibited the function of IP3Rs on ER membrane, since the majority of IP3Rs is expressed on ER membrane, but not on plasma membrane. Or a different protein on plasma membrane functions as a receptor for Chgb. In any case, we cannot emphasize the hypothesis. We have toned down to explain the hypothesis in Discussion (Page 26, Line 11-13).

3. All functional data are derived from primary cultures and coculture models. While these systems are well established and carefully executed, the lack of in vivo validation limits the translational impact. Given the strong disease relevance proposed, even limited in vivo confirmation (e.g., antibody treatment in an EAE model or tissue-level apoptosis analysis) would greatly enhance confidence in the conclusions.

We agreed that it is a very important point. However, it takes much time to set up these in vivo or ex vivo experiments and it was impossible to obtain a promising result during this revision period. We will try to perform these experiments in our next study. Instead, we have described this point as a limitation of this study and have toned down to explain the physiological role of Chgb in Discussion (Page 28, Line 8-17).

4. The authors show that TNF-α induces Chgb secretion from neurons and indirectly promotes oligodendrocyte apoptosis. Although they control for direct TNF-α toxicity in monoculture, TNF-α is a complex cytokine with multiple downstream effects, including the induction of other cytokines such as IL-6, IL-1β, IL-10, and TGF-β. A more detailed discussion of possible indirect pathways, beyond Chgb alone, would make the interpretation more balanced.

Thank you for your comment and advice. We have added descriptions of the effects of the cytokines on Chgb activity in Discussion (Page 28, Line 12-17).

5. The manuscript repeatedly suggests that anti-Chgb antibodies may represent a promising therapeutic strategy. While the in vitro neutralization data are convincing, the absence of in vivo efficacy and safety data makes this claim somewhat speculative. The authors may wish to tone down this aspect or frame it more clearly as a long-term possibility rather than an immediate translational outcome.

We agreed with your opinion. We have toned down to explain the possibility of anti-Chgb antibodies as therapeutic agents in the manuscript (Page 2, Line 16; Page 6, Line 4; Page 28, Line 8-9; Page 29, Line 6-8).

6. Several of the immunofluorescence panels, particularly in the main figures and supplementary data, are not consistently presented at a high technical standard. In multiple cases, the signal appears either weak or uneven, and in some images, the fluorescence seems oversaturated, potentially masking cellular details and affecting interpretability. Since these images form a major basis for the quantitative analyses, improving image acquisition and presentation (including appropriate exposure settings and dynamic range) would strengthen confidence in the data. Where possible, providing representative low- and high-magnification views and avoiding overexposed regions would further enhance the clarity and reproducibility of the findings.

Thank you for your comment. We have replaced and added several images in main and supplementary figures. In particular, we carefully selected the images that exhibit cellular morphology as clearly as possible, except for the images with nuclear markers, such as TUNEL and Olig2 staining (Figs. 1B, D, 3B, D, S1B, S2B, C, S7, and S8).

7. The authors could also highlight another angle of the scenario involving the gap junction intercellular communication proteins between neurons and oligodendrocytes. Recent advances suggest that astrocytes and oligodendrocytes interact with the expression of specific gap junction proteins, such as Cx43 and Cx47, respectively. These junctional proteins can be crucial for transmitting specific signals and for influencing the overall homeostasis of the CNS. This may be currently out of scope for the authors, but they could consider mentioning it as a future perspective in the discussion section. This will enhance the manuscript by highlighting another angle of transmission of Chgb within cells.

Thank you for the interesting information. We have added a speculative mechanism of Chgb functions in the intercellular networks through connexins in Discussion (Page 26, Line 15-18; Page 27, Line 1-4).

Minor Comments

1. The statistical section states that experiments were performed “at least 3 times,” but exact biological replicate numbers are not always clear in the figure legends. Providing precise n values for both technical and biological replicates would improve transparency.

Thank you for your advice. We have added to describe the number of the independent experiments (biological replicates) in each Figure legend (Page 16, Line 17-18; Page 18, Line 9-10; Page 18, Line 15-16; Page 20, Line 5-6; Page 21, Line 5-6; Page 21, Line 11-12; Page 23, Line 16; Page 24, Line 3-4; Page 24, Line 8-9). There is a variability in the number of independent experiments between the analyses, because in some of the experiments, all the prepared cells, including cells for controls, were already more differentiated when plated, or were less differentiated after induction of differentiation. These were probably due to the different timing of their differentiation between the independent experiments, while they were cultured in the mixed glial culture from different rat cerebral cortexes. The number of technical replicates was one in all the independent experiments since the cell number in each independent experiment from the mixed glial culture was not enough to prepare multiple technical replicants for all the experimental conditions. We have added this explanation in Materials and Methods (Page 15, Line 1-8).

2. There are minor typographical and formatting inconsistencies (for example, spacing around symbols and occasional line-break artifacts) that should be corrected during revision.

Thank you for letting us know. We have carefully corrected these parts throughout the text.

3. The Discussion could benefit from a clearer separation between experimentally supported conclusions and speculative hypotheses.

Thank you for your suggestion. However, it was difficult to clearly separate them, although we tried to do it as much as possible. We have described the experimentally supported conclusion in the first paragraph. The second and third paragraphs are more speculatively hypothetic sections of cellular and molecular mechanisms. The fourth paragraph contains the physiological relevance of Chgb in diseases and a limitation regarding its concentration as you mentioned above. In the fifth paragraph, we have described discussions about the cytokine effects and limitations of this study as you and Reviewer #2 suggested to discuss. The sixth paragraph is the summary section. We hope this shape of the Discussion sections would be accepted.

Response to Reviewer #2:

Thank you very much for your valuable comments. Below is our point-by-point response. Revised parts in the text are highlighted in yellow.

Reviewer #2: In this study, the authors investigated the effect of extracellular chromogranin B (Chgb) on oligodendrocyte lineage cells. The study aimed to explore and discuss the role of neuron-derived ChGB in remyelination in multiple sclerosis. While the study raises some interesting points, the experimental design and the results are unclear in many respects, which undermines the soundness of the study and the interpretation of the result

Major points

1. Line 207: 'OPCs were cultured in OPC medium for three days, and OLs were cultured in OL medium for three days'. Please provide a timeline indicating the complete process of culture preparation, including the stage-specific markers used in the study, as well as micrographs of the expected morphology. Please declare the DIV in each experiment.

Thank you for your suggestion. We have prepared a supplementary figure (S1 Fig), which indicates the timeline of the culture experiments and images of OPCs and OLs with their specific markers, NG2 and GalC, respectively, in the absence of Chgb. At DIV2, most of the cells were positive for NG2 with their bipolar morphology, which is typical for OPCs, while some cells were positive for GalC in their somas but not processes. At DIV4 of the OPC condition, most cells were still NG2-positive, although their morphology tended to change from bipolar to multipolar, which is typically observed in OPC culture on DIV4. At DIV5 of the OL condition, the majority in the cell populations was positive for GalC, but not for NG2. These GalC-positive cells formed the typical OL morphology with the highly branched cellular processes. We have added descriptions about this data in Materials and Methods (Page 10, Line 5-7).

2. The use of βIV-tubulin as an OPC marker expressed throughout the oligo lineage is highly questionable. Why not use NG2 or other markers that are clearly associated with the undifferentiated, proliferative stage?

In this study, we needed to perform the detergent treatment for cell permeability in TUNEL assay. In addition, we added the rabbit anti-Chgb antibody in the cultures for the inhibition assay. Therefore, it was necessary to use marker antibodies, which were detergent-resident and raised except in rabbit. We tested three antibodies to find the one that works well for the detection of OPCs in this specific condition. As a result, mouse anti-beta IV-tubulin antibody was most suitable among them. The mouse anti-beta IV-tubulin antibody also detected OLs in the condition as well. We have added images in S2 Fig and have added a description in Materials and Methods (Page 13, Line 16-18; Page 14, Line 1-2).

3. In the figures, PLP-positive cells exhibit different morphological phenotypes: quite immature in Fig. 1 and Fig. 3, and convincing as OLs in Fig. 4. Are the DIVs the same? If so, could the authors provide different micrographs or explain this difference?

Thank you for pointing it. We have replaced images of PLP-positive cells in Figs. 1D and 3D. These images were taken on DIV5 of the OL culture condition (see in S1 Fig), same as the images in Fig. 4B.

4. In the experimental design, the toxic effect of Chgb is tested under standard culture conditions. Since all the results are discussed in terms of inflammatory-demyelinating diseases and cells of the oligodendrocyte lineage are very sensitive to inflammatory cytokines in vitro, the study would benefit from investigating the effect of Chgb in the presence of inflammatory challenges and TNFα.

Thank you for your comment. We agreed that it is an important point. The expression of the cytokines, such as several interleukins, which are induced by TNF-α, is observed in astrocytes, microglia, and other activated immune cells. We are currently planning to set up in vivo and ex vivo experiments to analyze the effect of Chgb in the complex condition with the expression of the various inflammatory cytokines, as you pointed. However, it takes time to set up these experiments and it was impossible to obtain a convincing result during this revision period. We will try to perform these experiments in our next study. Instead, we have described these points as a limitation of this study and have toned down to explain the physiological role of Chgb in Discussion (Page 28, Line 8-17).

5. Co-culture: the authors used OPC/OL derived from the cerebral cortex and DRG-derived neurons. Although this is a very popular in vitro myelination assay, it is highly questionable given that Schwann cells are the myelinating cells of DRG axons. This must be discussed as a limitation of the study.

Thank you for pointing it. We have added to a description of the limitation in Discussion (Page 28, Line 18; Page 29, Line 1).

6. Figure 5 and co-culture experiment: the cells presented in Figure 5 seem to be OPCs, not OLs (as stated in line 209). This is because Olig2 is a transcription factor that regulates the very early stages of the oligodendrocyte lineage, and the morphology is consistent with that of OPCs. How long were the cells cultured for? This is a key point since OPCs and OLs play different roles in inflammatory-demyelinating conditions, albeit coherent ones. In this experiment, the author states that TNFα does not induce apoptosis in OL monocultures. This is quite surprising, given that extensive literature indicates TNFα as a major factor in OL degeneration

Attachment Submitted filename: 01) Response to Reviewers-2.docx

# Peer review: Decision Letter 1

**DOI:** 10.1371/journal.pone.0358362.r003

**Authors:** Eliseo Eugenin

27 May 2026

Dear Dr. Suzuki,

Thank you for submitting your manuscript to PLOS ONE. After careful consideration, we feel that it has merit but does not fully meet PLOS ONE’s publication criteria as it currently stands. Therefore, we invite you to submit a revised version of the manuscript that addresses the points raised during the review process.

==============================

**ACADEMIC EDITOR: Please insert comments here and delete this placeholder text when finished.**  Be sure to:

Dear Dr. Suzuki

Thank you for submitting your manuscript to PLOSone. Please answer all the concerns of the reviewers. If some concerns can not be addressed, please justify the point.

Best Regards

Eliseo Eugenin

==============================

Please submit your revised manuscript by Jul 11 2026 11:59PM. If you will need more time than this to complete your revisions, please reply to this message or contact the journal office at plosone@plos.org. When you're ready to submit your revision, log on to https://www.editorialmanager.com/pone/ and select the 'Submissions Needing Revision' folder to locate your manuscript file.

- A letter that responds to each point raised by the academic editor and reviewer(s). You should upload this letter as a separate file labeled 'Response to Reviewers'.
- A marked-up copy of your manuscript that highlights changes made to the original version. You should upload this as a separate file labeled 'Revised Manuscript with Track Changes'.
- An unmarked version of your revised paper without tracked changes. You should upload this as a separate file labeled 'Manuscript'.

If you would like to make changes to your financial disclosure, please include your updated statement in your cover letter. Guidelines for resubmitting your figure files are available below the reviewer comments at the end of this letter.

If applicable, we recommend that you deposit your laboratory protocols in protocols.io to enhance the reproducibility of your results. Protocols.io assigns your protocol its own identifier (DOI) so that it can be cited independently in the future. For instructions see: https://journals.plos.org/plosone/s/submission-guidelines#loc-laboratory-protocols. Additionally, PLOS ONE offers an option for publishing peer-reviewed Lab Protocol articles, which describe protocols hosted on protocols.io. Read more information on sharing protocols at https://plos.org/protocols?utm_medium=editorial-email&utm_source=authorletters&utm_campaign=protocols.

As the corresponding author, your ORCID iD is verified in the submission system and will appear in the published article. PLOS supports the use of ORCID, and we encourage all coauthors to register for an ORCID iD and use it as well. Please encourage your coauthors to verify their ORCID iD within the submission system before final acceptance, as unverified ORCID iDs will not appear in the published article. *Only*  the individual author can complete the verification step; PLOS staff *cannot*  verify ORCID iDs on behalf of authors.

We look forward to receiving your revised manuscript.

Kind regards,

Eliseo A Eugenin, Ph.D.

Academic Editor

PLOS One

Journal Requirements:

If the reviewer comments include a recommendation to cite specific previously published works, please review and evaluate these publications to determine whether they are relevant and should be cited. There is no requirement to cite these works unless the editor has indicated otherwise.

Additional Editor Comments:

Dear Dr. Suzuki

Thank you for submitting your manuscript to PLOSone. Please answer all the concerns of the reviewers. If some concerns can not be addressed, please justify the point.

Best Regards

Eliseo Eugenin

[Note: HTML markup is below. Please do not edit.]

Reviewers' comments:

Reviewer's Responses to Questions

**Comments to the Author**

Reviewer #1: (No Response)

Reviewer #2: (No Response)

**********

2. Is the manuscript technically sound, and do the data support the conclusions?

Reviewer #1: Partly

Reviewer #2: Yes

**********

3. Has the statistical analysis been performed appropriately and rigorously?

Reviewer #1: Yes

Reviewer #2: Yes

**********

4. Have the authors made all data underlying the findings in their manuscript fully available?

The PLOS Data policy

Reviewer #1: Yes

Reviewer #2: Yes

**********

5. Is the manuscript presented in an intelligible fashion and written in standard English?

Reviewer #1: Yes

Reviewer #2: Yes

**********

Reviewer #1: The revised manuscript is improved, and many of the earlier concerns have been addressed. However, an important issue remains regarding the mechanistic interpretation. The additional IP3R inhibitor experiments were specifically performed to support the proposed mechanism that extracellular Chgb induces apoptosis through IP3R-related signaling. However, the authors themselves state that these experiments did not support the hypothesis, since the inhibitor failed to produce statistically significant effects. This is an important negative result and should be treated more explicitly in the manuscript. At present, the study convincingly shows that recombinant and neuron-derived Chgb can induce apoptosis in OL-lineage cells in vitro, but the mechanistic basis remains unresolved. Similarly, the lack of in vivo or ex vivo validation means the direct relevance to demyelinating disease pathology remains speculative. These limitations do not invalidate the cell culture findings, but they substantially weaken the biological and translational conclusions.

The manuscript should avoid implying that the signaling pathway itself has been established. The absence of in vivo or ex vivo validation becomes more important in light of the unresolved mechanism. At present, the manuscript demonstrates a cell culture phenomenon but has not yet identified a validated pathological pathway in demyelinating disease. The therapeutic discussion regarding anti-Chgb antibodies should remain conservative, as the receptor mechanism and in vivo disease relevance remain unclear. Although several IF panels have improved, some microscopy images still appear uneven or partially oversaturated. A final careful review of image presentation would improve overall figure quality.

I have given a major revision with the understanding that these experiments are crucial for the current manuscript, and I have also submitted a comment to the editor regarding checking the journal policy and taking a stand on whether a major revision is required. If the new in vivo experiments and the other experiments cannot be added, the paper should be rewritten with the available results that align with the hypothesis.

Reviewer #2: All comments have been addressed. I would simply suggest to group the study limitations in a dedicated paragraph

**********

what does this mean?). If published, this will include your full peer review and any attached files.

If you choose “no”, your identity will remain anonymous but your review may still be made public.

**Do you want your identity to be public for this peer review?**  For information about this choice, including consent withdrawal, please see our Privacy Policy

Reviewer #1: No

Reviewer #2: **Yes:**  Laura Calza

**********

[NOTE: If reviewer comments were submitted as an attachment file, they will be attached to this email and accessible via the submission site. Please log into your account, locate the manuscript record, and check for the action link "View Attachments". If this link does not appear, there are no attachment files.]

To ensure your figures meet our technical requirements, please review our figure guidelines: https://journals.plos.org/plosone/s/figures

You may also use PLOS’s free figure tool, NAAS, to help you prepare publication quality figures: https://journals.plos.org/plosone/s/figures#loc-tools-for-figure-preparation.

NAAS will assess whether your figures meet our technical requirements by comparing each figure against our figure specifications.

# Author comment: Author response to Decision Letter 2

**DOI:** 10.1371/journal.pone.0358362.r004

12 Jun 2026

Response to Reviewer #1:

Thank you very much for your valuable comments. Below is our response. Revised parts in the text are highlighted in yellow.

Reviewer #1: The revised manuscript is improved, and many of the earlier concerns have been addressed. However, an important issue remains regarding the mechanistic interpretation. The additional IP3R inhibitor experiments were specifically performed to support the proposed mechanism that extracellular Chgb induces apoptosis through IP3R-related signaling. However, the authors themselves state that these experiments did not support the hypothesis, since the inhibitor failed to produce statistically significant effects. This is an important negative result and should be treated more explicitly in the manuscript. At present, the study convincingly shows that recombinant and neuron-derived Chgb can induce apoptosis in OL-lineage cells in vitro, but the mechanistic basis remains unresolved. Similarly, the lack of in vivo or ex vivo validation means the direct relevance to demyelinating disease pathology remains speculative. These limitations do not invalidate the cell culture findings, but they substantially weaken the biological and translational conclusions.

The manuscript should avoid implying that the signaling pathway itself has been established. The absence of in vivo or ex vivo validation becomes more important in light of the unresolved mechanism. At present, the manuscript demonstrates a cell culture phenomenon but has not yet identified a validated pathological pathway in demyelinating disease. The therapeutic discussion regarding anti-Chgb antibodies should remain conservative, as the receptor mechanism and in vivo disease relevance remain unclear. Although several IF panels have improved, some microscopy images still appear uneven or partially oversaturated. A final careful review of image presentation would improve overall figure quality.

I have given a major revision with the understanding that these experiments are crucial for the current manuscript, and I have also submitted a comment to the editor regarding checking the journal policy and taking a stand on whether a major revision is required. If the new in vivo experiments and the other experiments cannot be added, the paper should be rewritten with the available results that align with the hypothesis.

Thank you for your comment. As we explained in the last revision, it takes much time to set up the in vivo or ex vivo experiments and it was very difficult to obtain a promising result during this revision period. We will challenge these experiments, including experiments for the elucidation of the molecular mechanism, in our next study. We hope you could understand the situation.

We agree that we should not emphasize speculations about the molecular mechanism and the physiological relevance without experimental data. We have eliminated the paragraphs regarding the molecular mechanism and the possible physiological function from Discussion (2nd and 3rd paragraphs in the former version) (We have kept and moved the part described about gap junction proteins, which you suggested in the former revision: Page 27, Line 10-17). Instead, we have added a paragraph that summarize the available results and conclusion indicated by the results in Discussion (Page 24, Line 11-18; Page 25, Line 1-4). In addition, we have revised several sentences, which meant possible physiological and pathological functions of Chgb in Abstract, Introduction, and Discussion (Page 2, Line 14-15; Page 6, Line 1-2; Page 28, Line 1-3).

In terms of the oversaturated fluorescent images, we have replaced them with better ones (Figs. 1D, 3D, S1B, and S2A). We chose the images that are not oversaturated but show cellular morphology, including branched processes. We hope these images fit with the criteria. Thank you so much for your consideration.

Response to Reviewer #2:

Reviewer #2: All comments have been addressed. I would simply suggest to group the study limitations in a dedicated paragraph

We appreciate for your review and comment. We will work on the limitations you pointed in our next study.

Attachment Submitted filename: 01) Response to Reviewers-3.docx

# Peer review: Decision Letter 2

**DOI:** 10.1371/journal.pone.0358362.r005

**Authors:** Eliseo Eugenin

19 Jul 2026

Dear Dr. Suzuki

Thank you for submitting your manuscript to PLOS ONE. After careful consideration, we feel that it has merit but does not fully meet PLOS ONE’s publication criteria as it currently stands. Therefore, we invite you to submit a revised version of the manuscript that addresses the points raised during the review process.

Please submit your revised manuscript by Sep 02 2026 11:59PM. If you will need more time than this to complete your revisions, please reply to this message or contact the journal office at plosone@plos.org. When you're ready to submit your revision, log on to https://www.editorialmanager.com/pone/ and select the 'Submissions Needing Revision' folder to locate your manuscript file.

- A letter that responds to each point raised by the academic editor and reviewer(s). You should upload this letter as a separate file labeled 'Response to Reviewers'.
- A marked-up copy of your manuscript that highlights changes made to the original version. You should upload this as a separate file labeled 'Revised Manuscript with Track Changes'.
- An unmarked version of your revised paper without tracked changes. You should upload this as a separate file labeled 'Manuscript'.

If you would like to make changes to your financial disclosure, please include your updated statement in your cover letter. Guidelines for resubmitting your figure files are available below the reviewer comments at the end of this letter.

If applicable, we recommend that you deposit your laboratory protocols in protocols.io to enhance the reproducibility of your results. Protocols.io assigns your protocol its own identifier (DOI) so that it can be cited independently in the future. For instructions see: https://journals.plos.org/plosone/s/submission-guidelines#loc-laboratory-protocols. Additionally, PLOS ONE offers an option for publishing peer-reviewed Lab Protocol articles, which describe protocols hosted on protocols.io. Read more information on sharing protocols at https://plos.org/protocols?utm_medium=editorial-email&utm_source=authorletters&utm_campaign=protocols.

As the corresponding author, your ORCID iD is verified in the submission system and will appear in the published article. PLOS supports the use of ORCID, and we encourage all coauthors to register for an ORCID iD and use it as well. Please encourage your coauthors to verify their ORCID iD within the submission system before final acceptance, as unverified ORCID iDs will not appear in the published article. *Only*  the individual author can complete the verification step; PLOS staff *cannot*  verify ORCID iDs on behalf of authors.

We look forward to receiving your revised manuscript.

Kind regards,

Eliseo A Eugenin, Ph.D.

Academic Editor

PLOS One

Journal Requirements:

If the reviewer comments include a recommendation to cite specific previously published works, please review and evaluate these publications to determine whether they are relevant and should be cited. There is no requirement to cite these works unless the editor has indicated otherwise.

Additional Editor Comments:

Dear Dr. Suzuki

Thank you for submit answers to most of the comments. However, several still remain active.

Eliseo

[Note: HTML markup is below. Please do not edit.]

Reviewers' comments:

Reviewer's Responses to Questions

**Comments to the Author**

Reviewer #2: All comments have been addressed

Reviewer #3: (No Response)

**********

2. Is the manuscript technically sound, and do the data support the conclusions?

Reviewer #2: Yes

Reviewer #3: No

**********

3. Has the statistical analysis been performed appropriately and rigorously?

Reviewer #2: Yes

Reviewer #3: No

**********

4. Have the authors made all data underlying the findings in their manuscript fully available?

The PLOS Data policy

Reviewer #2: Yes

Reviewer #3: No

**********

5. Is the manuscript presented in an intelligible fashion and written in standard English?

Reviewer #2: Yes

Reviewer #3: Yes

**********

Reviewer #2: All comments have been addressed, including the indication of the study limits. The manuscript is new acceptable in the present form

Reviewer #3: The authors have made an effort to address some of the previous comments, particularly by toning down speculative molecular mechanisms. However, this revised version still suffers from major methodological flaws, severe internal statistical contradictions, and a lack of technical rigor in both primary culture descriptions and image acquisition. Crucially, the authors' justification for cell type identification and experimental design remains scientifically unsound.

Major Concerns

1. In lines 228-232, the authors state that they used a mouse anti-beta-IV-tubulin antibody as a surrogate marker to quantify both OPCs and mature OLs due to species constraints and TUNEL assay requirements. This approach is scientifically incorrect. Established literature (e.g., Terada et al., Glia, 2005) clearly demonstrates that beta-IV-tubulin is exclusively expressed in pre-myelinating and mature, differentiated oligodendrocytes, while being virtually absent in undifferentiated, proliferating OPCs. This indicates a technical failure of their immunocytochemistry protocol or antibody batches in their laboratory, which cannot be bypassed by mischaracterizing a mature cytoskeletal marker (beta-IV-tubulin) as an OPC marker.

Furthermore, the morphology of the cells in some images, as at DIV4 in Fig S2A, is highly branched and stellate, typical of differentiating OLs, and fundamentally different from the simple bipolar/tripolar morphology of true OPCs correctly shown at DIV1 (Fig S1B DIV1). This terminology must be corrected, and the OPC death data must be re-evaluated using functional, specific progenitor markers.

2. In lines 271-272, the authors state that the number of OPCs significantly decreased in the presence of 5 μg/mL mChgb, but the effect did not reach statistical significance at 20 μg/mL. This is a fatal internal contradiction. If a higher concentration of a toxic factor yields a non-significant reduction in cell numbers compared to a lower concentration, the claim of a specific, dose-dependent induction of apoptosis is invalidated. This contradiction is further exacerbated by the TUNEL results (lines 292-294): the authors report significant TUNEL-positive ratios at 20 μg/mL (where cell loss was non-significant) but do not show significance at 5 μg/mL (where cell loss was significant). A significant reduction in cell numbers paired with a negative TUNEL assay strongly points toward acute cell lysis/necrosis (where dead cells detach and disappear from the field, as qualitatively observed in the nearly empty fields of Fig 2A at 5 μg/mL) rather than regulated apoptosis.

3. The authors state that the neuron-OPC/OL co-cultures were maintained for only 5 days (lines 201-206; 212-213). To justify this co-culture setup, the authors cite established literature in the field. However, the cited literature utilizes a rigorous 14-day co-culture protocol to allow proper axonal outgrowth, alignment, and robust myelination wrapping. A 5-day co-culture represents an extremely immature and unstable system where cells are still adapting to the media transition. The authors must explain why they drastically shortened this validated 14-day protocol and how they can exclude that the observed apoptotic effects at 5 DIV are mere artifacts of an unestablished culture system.

4. The Materials and Methods section completely lacks a protocol or citation describing how primary OPCs were isolated from neonatal rats. Similarly, under "Preparation of primary neurons" (line 179) (which should be more accurately titled "Primary DRG neurons") the text completely omits the isolation methodology (enzymatic digestion, tissue dissociation, centrifugation, etc.). It merely states that DRGs were "collected and plated."

5. For the Western Blot analysis of Chgb in the conditioned media of DRG neurons (lines 376-378), the authors do not state how the media was processed. Was the media concentrated (e.g., via TCA precipitation or centrifugal filters)? How was the loading volume normalized (by total cell protein, cell count, or initial volume)? This information is mandatory for reproducibility.

6. The technical quality of the immunofluorescence panels (particularly for TUNEL in Fig 2 and Cleaved Caspase-3) is suboptimal. The green channels for both TUNEL and CC3 exhibit heavy pixelated noise, high background grain, and exceptionally weak, diffuse signals indicated by the arrows. Real apoptotic markers should display intense, compact nuclear or peri-nuclear signals that perfectly co-localize with condensed DAPI nuclei.

7. Moreover, the authors omitted any mention of a permeabilization agent (such as Triton X-100) in their Immunocytochemistry section (lines 214-222). Because CC3, TUNEL enzymes, and beta-IV-tubulin require access to internal cellular compartments, the protocol as written is incomplete.

8. In the neutralizing antibody assays (lines 316-317), the authors suddenly shifted the experimental conditions to 10 μg/mL of mChgb combined with 10 μg/mL of anti-Chgb antibody. Given that the main toxicity assays were performed at 5 and 20 μg/mL, why did the authors choose an arbitrary intermediate dose for the inhibition assay? Furthermore, the protocol details of this inhibition (e.g., whether the antibody was pre-incubated with the recombinant protein prior to cell treatment, and the precise molar ratio) must be provided.

Minor Concerns

1. Lines 60-62: The statement regarding the higher degeneration of myelinated vs. unmyelinated axons in MS and EAE requires appropriate citations.

2. Lines 65-66: The claim that neurons utilize an intrinsic clearance mechanism to remove dysfunctional myelin by releasing factors that induce OL death requires a reference.

3. In the Introduction, the authors imply that Chgb is exclusively released by neurons. Under neuroinflammatory conditions (such as MS/EAE), reactive astrocytes and microglia are also known to express and modulate secretogranins/chromogranins. This should be discussed more accurately.

4. In accordance with PLOS ONE guidelines for transparency and scientific rigor, the authors must provide the commercial catalog for all primary antibodies, secondary antibodies, and key kits (e.g., Power Block, TUNEL kit).

5. Lines 209-213 (OPCs were cultured... mChgb was added...) describe cell culture timelines and treatments. This text does not belong under the "Immunocytochemistry" subheading and should be moved to the appropriate primary culture methodology section.

6. The authors state they used Student's t-test and One-way ANOVA but did not mention performing any preliminary tests for normal distribution (e.g., Shapiro-Wilk test). This is mandatory, especially since they note that "independent experiments were added when variability was high" (lines 255-256).

7. The authors must report the exact microscopy metadata (microscope model, software, objective magnification, and numerical aperture) used to capture the images. They should also specify how many coverslips were analyzed per independent experiment and how many fields were randomly selected (if it is) and counted per coverslip.

8. In lines 453-456, the authors attempt to link high Chgb expression in healthy brain areas (hippocampus, amygdala) to Alzheimer's Disease (AD) via a tenuous connection to myelin. Since Chgb is high in healthy individuals in these regions, this logical leap is highly speculative and irrelevant to their acute oligodendrocyte apoptosis model. This paragraph should be removed or completely reframed.

**********

what does this mean?). If published, this will include your full peer review and any attached files.

If you choose “no”, your identity will remain anonymous but your review may still be made public.

**Do you want your identity to be public for this peer review?**  For information about this choice, including consent withdrawal, please see our Privacy Policy

Reviewer #2: No

Reviewer #3: No

**********

[NOTE: If reviewer comments were submitted as an attachment file, they will be attached to this email and accessible via the submission site. Please log into your account, locate the manuscript record, and check for the action link "View Attachments". If this link does not appear, there are no attachment files.]

To ensure your figures meet our technical requirements, please review our figure guidelines: https://journals.plos.org/plosone/s/figures

You may also use PLOS’s free figure tool, NAAS, to help you prepare publication quality figures: https://journals.plos.org/plosone/s/figures#loc-tools-for-figure-preparation.

NAAS will assess whether your figures meet our technical requirements by comparing each figure against our figure specifications.

# Author comment: Author response to Decision Letter 3

**DOI:** 10.1371/journal.pone.0358362.r006

27 Aug 2026

Comment from the academic editor:

Thus, only add the comments in the discussion, like limitations or in future experiments.

Response to the editor’s comment:

Thank you very much for your comment. We have revised a paragraph about limitations of this study and future experiments in Discussion (Page 26, Line 14-18; Page 27, Line 7-9). Revised parts in the text are highlighted in yellow.

Attachment Submitted filename: 1) Response to Reviewers-4.docx

# Editor report: Decision Letter 3

**DOI:** 10.1371/journal.pone.0358362.r007

**Authors:** Eliseo Eugenin

31 Aug 2026

Chromogranin B acts as a neuronal paracrine factor to trigger oligodendrocyte apoptosis

PONE-D-25-68487R3

Dear Dr. Suzuki,

We’re pleased to inform you that your manuscript has been judged scientifically suitable for publication and will be formally accepted for publication once it meets all outstanding technical requirements.

Within one week, you’ll receive an e-mail detailing the required amendments. When these have been addressed, you’ll receive a formal acceptance letter and your manuscript will be scheduled for publication.

An invoice will be generated when your article is formally accepted. Please note, if your institution has a publishing partnership with PLOS and your article meets the relevant criteria, all or part of your publication costs will be covered. Please make sure your user information is up-to-date by logging into Editorial Manager at Editorial Manager® and clicking the ‘Update My Information' link at the top of the page. For questions related to billing, please contact billing support.

If your institution or institutions have a press office, please notify them about your upcoming paper to help maximize its impact. If they’ll be preparing press materials, please inform our press team as soon as possible -- no later than 48 hours after receiving the formal acceptance. Your manuscript will remain under strict press embargo until 2 pm Eastern Time on the date of publication. For more information, please contact onepress@plos.org.

Kind regards,

Eliseo A Eugenin, Ph.D.

Academic Editor

PLOS One

Additional Editor Comments (optional):

Dear Dr. Suzuki

Thank you for providing the changes required.

Best Regards

Eliseo Eugenin

Reviewers' comments:

# Editor report: Acceptance letter

**DOI:** 10.1371/journal.pone.0358362.r008

**Authors:** Eliseo Eugenin

PONE-D-25-68487R3

PLOS One

Dear Dr. Suzuki,

I'm pleased to inform you that your manuscript has been deemed suitable for publication in PLOS One. Congratulations! Your manuscript is now being handed over to our production team.

At this stage, our production department will prepare your paper for publication. This includes ensuring the following:

* All references, tables, and figures are properly cited

* All relevant supporting information is included in the manuscript submission,

* There are no issues that prevent the paper from being properly typeset

You will receive further instructions from the production team, including instructions on how to review your proof when it is ready. Please keep in mind that we are working through a large volume of accepted articles, so please give us a few days to review your paper and let you know the next and final steps.

Lastly, if your institution or institutions have a press office, please let them know about your upcoming paper now to help maximize its impact. If they'll be preparing press materials, please inform our press team within the next 48 hours. Your manuscript will remain under strict press embargo until 2 pm Eastern Time on the date of publication. For more information, please contact onepress@plos.org.

You will receive an invoice from PLOS for your publication fee after your manuscript has reached the completed accept phase. If you receive an email requesting payment before acceptance or for any other service, this may be a phishing scheme. Learn how to identify phishing emails and protect your accounts at https://explore.plos.org/phishing.

If we can help with anything else, please email us at customercare@plos.org.

Thank you for submitting your work to PLOS One and supporting open access.

Kind regards,

PLOS One Editorial Office Staff

on behalf of

Dr. Eliseo A Eugenin

Academic Editor

PLOS One
